# Restriction of access to the central cavity is a major contributor to substrate selectivity in plant ABCG transporters

**DOI:** 10.1007/s00018-023-04751-6

**Published:** 2023-03-23

**Authors:** Konrad Pakuła, Carlos Sequeiros-Borja, Wanda Biała-Leonhard, Aleksandra Pawela, Joanna Banasiak, Aurélien Bailly, Marcin Radom, Markus Geisler, Jan Brezovsky, Michał Jasiński

**Affiliations:** 1 Department of Plant Molecular Physiology, Institute of Bioorganic Chemistry, Polish Academy of Sciences, Z. Noskowskiego 12/14, 61-704 Poznan, Poland; 2 NanoBioMedical Centre, Adam Mickiewicz University, Wszechnicy Piastowskiej 3, 61-614 Poznan, Poland; 3 Laboratory of Biomolecular Interactions and Transport, Department of Gene Expression, Institute of Molecular Biology and Biotechnology, Faculty of Biology, Adam Mickiewicz University, Uniwersytetu Poznanskiego 6, 61-614 Poznan, Poland; 4 International Institute of Molecular and Cell Biology in Warsaw, Ks. Trojdena 4, 02-109 Warsaw, Poland; 5 Department of Plant and Microbial Biology, University of Zurich, Zollikerstrasse 107, 8008 Zurich, Switzerland; 6 Department of Structural Bioinformatics, Institute of Bioorganic Chemistry, Polish Academy of Sciences, Z.Noskowskiego12/14, 61-704 Poznan, Poland; 7 Institute of Computing Science, Poznan University of Technology, Piotrowo 2, 60-965 Poznan, Poland; 8 Department of Biology, University of Fribourg, Chem. du Musée 10, 1700 Fribourg, Switzerland; 9 Department of Biochemistry and Biotechnology, Poznan University of Life Sciences, Dojazd 11, 60-632 Poznan, Poland

**Keywords:** ABC transporters, AlphaFold2, Selectivity, Phenylpropanoids, Access path

## Abstract

ABCG46 of the legume *Medicago truncatula* is an ABC-type transporter responsible for highly selective translocation of the phenylpropanoids, 4-coumarate, and liquiritigenin, over the plasma membrane. To investigate molecular determinants of the observed substrate selectivity, we applied a combination of phylogenetic and biochemical analyses, AlphaFold2 structure prediction, molecular dynamics simulations, and mutagenesis. We discovered an unusually narrow transient access path to the central cavity of MtABCG46 that constitutes an initial filter responsible for the selective translocation of phenylpropanoids through a lipid bilayer. Furthermore, we identified remote residue F562 as pivotal for maintaining the stability of this filter. The determination of individual amino acids that impact the selective transport of specialized metabolites may provide new opportunities associated with ABCGs being of interest, in many biological scenarios.

**Supplementary Information:**

The online version contains supplementary material available at 10.1007/s00018-023-04751-6.

## Introduction

ATP-binding cassette (ABC) transporters are prominent proteins that translocate molecules through biological membranes using ATP as a source of energy [1]. Members of the ABC family are common in all domains of life, but plant genomes are exceptionally rich in genes encoding them [2, 3]. Taking into account their structure and phylogenetic relationships, most ABC proteins have been classified into eight subfamilies, designated ABCA-ABCH [4]. The particularly numerous plant ABC transporters translocate diverse molecules, such as lipids, phytohormones, carboxylates, heavy metals, chlorophyll catabolites, and xenobiotic conjugates, across various biological membranes [5]. In this manner, they participate in diverse processes, including organ growth, nutrition, development, responses to abiotic stresses, as well as both symbiotic and antagonistic relationships [2, 6, 7].

Especially abundant in plants are genes encoding so-called full-size ABCG transporters. A full-size ABCG transporter is a single polypeptide forming two transmembrane domains (TMDs), which constitute a membrane-spanning region, and two cytosolic domains called nucleotide-binding domains (NBDs). Depending on the transporter subfamilies, single TMD has five to ten transmembrane α-helices. Full-size members of the G family are characteristic for plants and fungi and distinct in their reverse organization of domains in the subunits namely NBD1-TMD1-NBD2-TMD2 [4, 8]. Moreover, in comparison to thoroughly studied ABCB proteins, where α-helices-forming TMDs represent a so-called domain swap arrangement, ABCG transporters revealed a different TMD organization, in which not individual helices but the entire TMD rotates as a solid body during the transport of molecules [9]. Full-size ABCGs were initially identified in *Saccharomyces cerevisiae* and the clinically relevant fungus, *Candida albicans.* They were described as pleiotropic drug resistance (PDR) proteins because they can act as efflux pumps, removing diverse molecules from these unicellular organisms, including exogenously applied drugs used in medical treatments, thus conferring resistance against large sets of chemicals [10]. Consequently, PDR proteins have attracted interest of biotechnologists and medical scientists. However, despite strenuous efforts, we still have limited understanding of molecular bases of their action.

Characterized plant full-size ABCGs include proteins that can transport several molecules that are not necessarily related but are usually endogenous metabolites. For instance, ABCG37 of *Arabidopsis thaliana* is involved in translocation of the auxin precursor, indolyl-3-butyric acid (IBA) [11], and the phenolic compound scopoletin [12, 13]. Arabidopsis ABCG36/PEN3/PDR8 transports, among others, IBA, the Brassicales-specific phytoalexin camalexin, heavy metals, and possibly monolignols [14–21]. However, despite initially considered as functional homologs of yeast multidrug pumps, at least certain plant full-size ABCG proteins appear to be selective toward translocated molecules. The specialization is proposed to be a consequence of a sophisticated chemodiversity exemplified by specialized metabolism that requires tightly controlled distribution of metabolites via dedicated transporters [3, 5].

Phenylpropanoids are a large class of specialized plant metabolites with many important roles in plant biology, medical applications, and industrial uses [22]. ABCG46 (formerly known as ABCG10) of the legume *Medicago truncatula*, which is required for efficient de novo production of the phenylpropanoid-derived phytoalexin medicarpin, selectively translocates 4-coumarate and liquiritigenin. Notably, structurally similar phenylpropanoids like naringenin, isoliquiritigenin, and 7,4′-dihydroxyflavone are not transported by MtABCG46 [23, 24].

Despite the recent progress in structural research regarding ABCG proteins [25–29], progressing from static atomic structures to an understanding of molecular mechanisms behind the substrate recognition and transport has been challenging [30]. This is partly because of the difficulty in obtaining experimental data at atomic detail, but also because of the lack of efficient sampling of the intricate process of a complete transport cycle. Fortunately, AlphaFold2 [31] has proven utility for addressing the first of these problems by providing accurate 3D structures of proteins from the amino acid sequence, even for large transmembrane hydrophobic proteins that are difficult to crystalize [32]. Structurally, although the complete transport process is still elusive, it is accepted that ABCG exporters start this process with an inward-facing (IF) conformation that allows substrate migration to the central cavity. The protein then undergoes large structural rearrangements to an outward-facing (OF) conformation, enabling release of the substrate to the extracellular environment. Binding, hydrolysis, and release of ATP as well as substrate recognition followed by its migration contribute to the intricacy of the overall transport process [28, 29].

So far, due to its clinical importance—as connected to the multidrug resistance—a non-selective transporter HsABCG2 is one of the most studied proteins from the G subfamily. Obtained HsABCG2 cryo-EM data provide some structure–function insights. For instance, it enabled for the identification of the short loop after TMD helix 5 called the valve/plug. The latter is proposed as an important structural element regulating conformation-dependent substrate release from the central (binding) cavity and preventing substrate reflux [29, 33]. Furthermore, previous simulation work has highlighted the valve region as crucial for substrate binding, structurally dividing the protein interior into two cavities, which are accessible at different stages of the transport process and permeable for at least some substrates with energy costs not greater than hydrolysis of an ATP molecule [33–35]. Also, highly conserved residues located in TMD helix 2 were found to be vital for trapping the substrate within the central cavity [36]. However, the molecular details behind the substrate recognition still remain unclear, as well as whether those are analogous within the structurally and functionally diverse group of ABCG transporters.

Using a combination of phylogenetic and biochemical analyses, AlphaFold2 structure prediction, molecular dynamics simulations, and mutagenesis, we have identified a transient access path in MtABCG46 that is directly involved in the recognition and passage of 4-coumarate and liquiritigenin through the plasma membrane. Moreover, we have identified F562 as a critical residue for the architecture of this access path responsible for selective transport.

## Materials and methods

### Plant material

*Nicotiana tabacum* Bright Yellow 2 (BY2) suspension cell cultures [37] were grown in Murashige and Skoog medium supplemented with 2.72 mM KH_2_PO_4_, 0.56 mM myoinositol, 3 μM thiamine, 0.9 μM 2,4-dichlorophenoxyacetic acid, and 87.64 mM sucrose, in the dark at 26 °C on an orbital shaker (130 rpm), and diluted 1:5 every week.

### Genetic constructs

All genetic constructs were based on the pMDC43 vector, carrying a GFP tag sequence [38]. p35S::GFP-MtABCG46 construct as well as particular mutants of p35S::GFP-MtABCG46 were generated by GenScript. All plasmid constructs were confirmed by DNA sequencing.

### Plant transformation

Stably transformed BY2 cells were generated by co-cultivation of 5-day-old BY2 suspension cells with *Agrobacterium tumefaciens* strain AGL1 [39] carrying the pMDC43 vector containing a particular variant of the MtABCG46 sequence or the empty pMDC43 vector, as previously described [24].

### Confocal microscopy

Five-day-old suspension cell cultures overexpressing GFP-fused MtABCG46 variants were observed by laser scanning confocal microscopy (with a Leica TCS SP5 AX v.2.7 instrument). Plasma membranes of sampled cells were stained with FM4-64 (ThermoFisher Scientific) according to the manufacturers’ protocol, no fixation was applied. Obtained pictures were analyzed using Leica LAS AF software. Fluorescent signals from GFP and FM4-64 were pseudo-colored in green and magenta, respectively. The excitation wavelength for GFP and FM4-64 was 488 nm. Fluorescence signals were collected at 485–547 nm (GFP) and 570–650 nm (FM4-64).

### Preparation of plasma membrane vesicles

Microsomal fractions were isolated from 12 g portions of BY2 suspension cell cultures as previously described [40]. Plasma membrane fractions of the microsomal isolates were enriched by partitioning in an aqueous two-phase system, also as previously described [41]. The quality of obtained microsomes was tested with 9-amino-6-chloro-2-methoxyacridine (ACMA; Invitrogen A1324) fluorescence quenching assays.

### Transport analysis with plasma membrane vesicles

The transport of phenolic compounds uptake was studied by the rapid filtration technique with 4-coumarate, liquiritigenin, isoliquiritigenin, 7,4′-dihydroxyflavone, and the plasma membrane microsomes using nitrocellulose filters (0.45 mm pore-size; Millipore). The transport assays were performed with microsomes corresponding to 520 ng μL^−1^ protein concentration mixed with transport buffer (10 mM Tris–HCl, 10 mM EDTA, 10% sucrose, pH 5.0), the selected phenolic (750 μM), 100 μg mL^−1^ creatine kinase, 10 mM creatine-phosphate, and 1 mM of MgCl_2_, in the presence and absence of 4 mM ATP. After 3 min incubation at 24 °C, 0.3 mL of each reaction mixture was immediately loaded on a prewetted filter and rapidly washed with 10 mL of ice-cold transport buffer. The filters were air dried for an hour, and then incubated in 80% MetOH with 0.1% formic acid. Phenolic compounds were extracted by adding chloroform:water mixture (1:0.25 sample volume) to the sample and centrifugation for 30 min in 13,200 rpm. The dried samples were dissolved in 80% methanol and subjected to HPLC/MS analyses, as described below. For competition assays, tested molecules were added, each at 750 μM, together to the transport buffer. Experiments were repeated three times with independent vesicle preparations unless stated otherwise.

### HPLC/MS analysis

Samples were analyzed by liquid chromatography–electrospray ionization–tandem mass spectrometry (LC/ESI/MS) using a Waters UPLC Acquity system, equipped with a C18 RP column, connected to a Bruker micrOTOF-Q II mass spectrometer. The mobile phase consisted of a gradient of 0.5% formic acid (v/v) in water (A) and 0.5% formic acid (v/v) in acetonitrile (B). The *m*/*z* range of the recorded MS spectra was 50–1000. The MS was operated in positive and negative ion modes for phenolics and carboxylic acids, respectively.

### Multiple sequence alignment and data filtering

To select full-size ABCG from multiple groups of plants, we subjected One Thousand Plant Transcriptomes (1KP) transcript data (https://db.cngb.org/onekp/) [42, 43] to tBLASTn searches using MtABCG46 as a query sequence with an E-value cutoff of 1e−5. Next, 26,889 1KP samples longer than 1000 bp were translated into six frames using software written in Visual C#. The longest ORFs starting with the ATG codon, after excluding the duplicates, were selected and further verified, by a BLASTp search against known ABC transporters belonging to different subfamilies was conducted. One thousand eight hundred and thirty-nine 1KP samples were assigned to a full-size ABCG subfamily with over 70% coverage and an E-value of 0.0, and used for the subsequent analysis. Multiple sequence alignment (MSA) of the full-size ABCG amino acid sequences was performed using the MUSCLE algorithm [44] in MEGA X [45]. For conservation analysis, complete alignment of predicted amino acid sequences of 1576 plant full-size ABCG transporters was submitted to the ConSurf server with default settings [46, 47]. We also submitted extracted taxa-specific alignments (66, 205, 91, 388, and 725—green alga, bryophyte, pteridophyte, monocots, and core eudicot sequences, respectively) to the ConSurf server, and subjected the complete alignment (1576 sequences) to co-evolution analysis using the Gremlin server [48, 49] with default settings.

### Modeling of the 3D structure

The amino acid sequence of the ABCG46 transporter of *Medicago truncatula* was obtained from the UniProt database (accession no. A0A396JDZ5), and submitted to a local installation of AlphaFold2 v2.1.0 using the default settings [31]. The resulting models were evaluated using the pLDDT score [31] and PROCHECK software [50].

To define positions of the Mg^2+^ ions, experimental structures of ABCG2 from *Homo sapiens* (PDB ID: 6hbu) and Pdr5 from *Saccharomyces cerevisiae* (PDB ID: 7p06) were employed as templates. The NBD region of the model and experimental structures were superimposed using TM-align software [51], and positions of the Mg^2+^ ions were copied to the model. For the ATP molecules, the NBD regions were also superimposed, but coordinates of the ATP atoms were not used directly. Instead, a docking box enclosing the molecules was built, then AutoDock Vina v1.1.2 [52] was used to determine their most suitable positions. Mutations at residue F562 to alanine, leucine, and tyrosine were performed with the tleap module of the Amber20 package [53], and one system of the wild-type protein was built without ATP and Mg^2+^ ions as an apo variant.

### Molecular dynamics (MD) simulations

The protein model was protonated with the H++ server [54] at pH 7.0. The protonated protein was embedded in a 1-palmitoyl-2-oleoyl-sn-glycero-3-phosphocholine (POPC) lipid bilayer using the CHARMM-GUI server with integrated PPM 2.0 method to obtain the system's coordinates in PDB format [55–59]. For the system, the size of the simulation box was set to 130 Å, with water thickness of 15 Å, and KCl salt concentration of 0.1 M. Further ATP molecules and Mg^2+^ ions were inserted as described above and the PDB file was converted to AMBER parameters and topology using tleap module of Amber20 package [53]. The water model employed was OPC [60], and the HMR [61] method was used to enable 4 fs simulation timesteps. The MD engine employed was Amber20 [53] with the pmemd GPU implementation [62], the ff19SB force field [63] was used for the protein, lipid17 for the POPC membrane, and previously presented parameters for the ATP molecules [64]. The system was minimized with 2500 steps of steepest descent, followed by 2500 steps of conjugated gradient, applying only positional restraints to the protein, ATP, and Mg^2+^ ions, but positional and dihedral restraints to the membrane (Supplementary Table 1). After the minimization, a series of 1 ns NVT and NPT equilibration simulations with Langevin thermostat and Monte Carlo barostat used as appropriate was employed to release restraints applied on the system (Supplementary Table 1). After the equilibration stage, an extra 100 ns NPT equilibration simulation was applied in which restraints were only maintained for the Mg^2+^ and ATP molecules (Supplementary Table 1). Finally, 100 ns unrestrained NPT simulation to fully equilibrate the system was performed, followed by a production phase of 400 ns unrestrained NPT simulation (Supplementary Table 1). For every variant considered, five replicas of the production phase were simulated.

To simulate conversion to more open IF conformation, umbrella sampling (US) [65] was employed on all protein variants with two aims: to obtain the structures in their IF-open conformations and study the dynamics in that conformation, and to evaluate the energetic cost of the opening process. Both processes employed the same procedure and input structures, varying in the biasing potential and simulation time for each umbrella. The open ScPDR5 structure with bound rhodamine 6G substrate was used as the target (PDB ID: 7p05). The US simulations were performed with the Amber20 package, using the RMSD to backbone atoms of the transmembrane helices of the target as the collective variable (Supplementary Fig. 1). For each variant, the US simulations were initiated from the structures exhibiting the lowest RMSD to the backbone atoms of the transmembrane helices of the target. Following, the ATP molecules of each system were replaced by ADP molecules present in the target structure, and the parameters were modified accordingly [64]. The systems were minimized with 1000 steps of steepest descent, followed by 1000 steps of conjugated gradient. Next, 250 ps of equilibration with NPT simulation were performed at 303.15 K and 1 bar using Langevin thermostat and Monte Carlo barostat. The US protocol to obtain IF-open structures consisted of 22, 21, 22, and 24 simulation windows for WT, F562L, F562Y, and F562A, respectively, equidistantly positioned (step 0.1 Å RMSD) along the collective variable until the target RMSD of 0 Å. For each window, a 1 ns NPT simulation was performed at 303.15 K and 1 bar using the Langevin thermostat and Monte Carlo barostat and the force constant of 1000 kcal mol^−1^ Å^−2^. At the end of the US runs, the final structures for each variant were used in MD simulations that followed the same simulation protocol described earlier. For each variant, 100 ns of MD without restraints was performed as the equilibration stage, followed by five replicates with 400 ns of unrestrained MD as the production stage. To evaluate the energetic cost of transition from IF-closed to IF-open states in each variant, three opening simulations were performed with 1, 2, and 4 ns of simulation time per window. In these US simulations, the RMSD step for each window was reduced to 0.02 Å to guarantee sufficiently large overlaps between windows for later analysis, using the following force constants: 500 kcal mol^−1^ Å^−2^, and increased to 1000 kcal mol^−1^ Å^−2^ from 1.7 to 1.40 Å RMSD, resulting in 36, 31, 36, and 46 simulation windows for WT, F562L, F562Y, and F562A, respectively. The potential of mean force was calculated with the weighted histogram analysis method [66] implemented by Grossfield, in version 2.0.11 [67] (http://membrane.urmc.rochester.edu/?page_id=126).

### Access path detection, classification, and selection

For path computation and detection, CAVER v3.0 software [68] was employed with a 0.9 Å probe radius, 6 Å shell radius, and 4 Å shell depth. For clustering, the average-link hierarchical algorithm was used, the maximum number of clusters was set to 50, and clustering threshold to 3.5. The starting position of the CAVER calculation was set to employ the center of mass of residues 562, 566, 1213, and 1217. Subsequently, TransportTools software v0.9.0 [69] was used to obtain a comprehensive and comparative view of the path network across all variants sourced from all converged CAVER calculations. In TransportTools, the clustering method was set to complete with a clustering cutoff of 0.5 Å for the analysis of the wild-type protein, then switched to the average method for the comparison of all variants in IF-closed and IF-open states simultaneously using clustering cutoffs of 2.0 and 0.5 Å, respectively, while all other parameters were left as default. The candidate selected as the correct access path was further sorted by the bottleneck radius and the 100 widest paths of each variant were used in the analysis and further ligand migration experiments.

### Ligand migration and energy barrier calculation

To study the capability of each variant to transport the four tested phenolic compounds, molecular docking across selected paths was performed with CaverDock software v1.1 [70]. 3D structures of the ligands of the four compounds were obtained from the PubChem database: liquiritigenin (CID 114829), isoliquiritigenin (CID 638278), 4-coumarate (CID 637542), and 7,4′-dihydroxyflavone (CID 5282073), then the MGLTools v1.5.6 [71] was used to prepare the files for CaverDock with the prepare_ligand4.py script with the default settings. In the same way, all the snapshots of the MDs from the variants where a path was selected were processed with the prepare_receptor4.py script with default settings. The exhaustiveness for CaverDock runs was set to one, using a single CPU core per task whereas eight parallel workers unique to CaverDock algorithm were used to enable extensive sampling of each path segment [70]. Finally, the upper-bound trajectories describing continuous ligand migration were analyzed with in-house Python scripts.

### Auxiliary analyses of data from MD simulations

Using the cpptraj [72] module of the Amber20 package, initially the heavy atoms’ root mean square deviation (RMSD) and the root mean square fluctuation (RMSF) of the whole system were calculated. However, since the main focus was the TMD region, separate RMSDs for specific regions were also calculated (Supplementary Fig. 2b and Supplementary Table 2). For the membrane, the lipid order parameters of lipid tails were evaluated together with the mass density with cpptraj to ensure the proper equilibrium behavior of the POPC membrane. To evaluate the behavior of the TMD helices forming the candidate access path, α helices 2, 5, 8, and 11 were analyzed with the HELANAL module of MDAnalysis v2.0.0 [73, 74].

### Statistical analyses

Statistical analyses were performed using GraphPad Prism software v9.0. The normality of distribution assumption was assessed for particular groups of values by Anderson–Darling, Shapiro–Wilk, and Kolmogorov–Smirnov normality tests. If data met normal distribution criteria, an unpaired *t* test with Welch's correction was applied*.* If data did not meet normal distribution criteria, non-parametric tests were applied (the two-tailed Mann–Whitney test or Kruskal–Wallis test with post hoc Dunn’s multiple comparison test). *P* values obtained can be found in the Supplementary Statistical Data.

## Results

### The MtABCG46 model features an unusually occluded central cavity connected with the intracellular environment by a transient access path

**Fig. 1:**
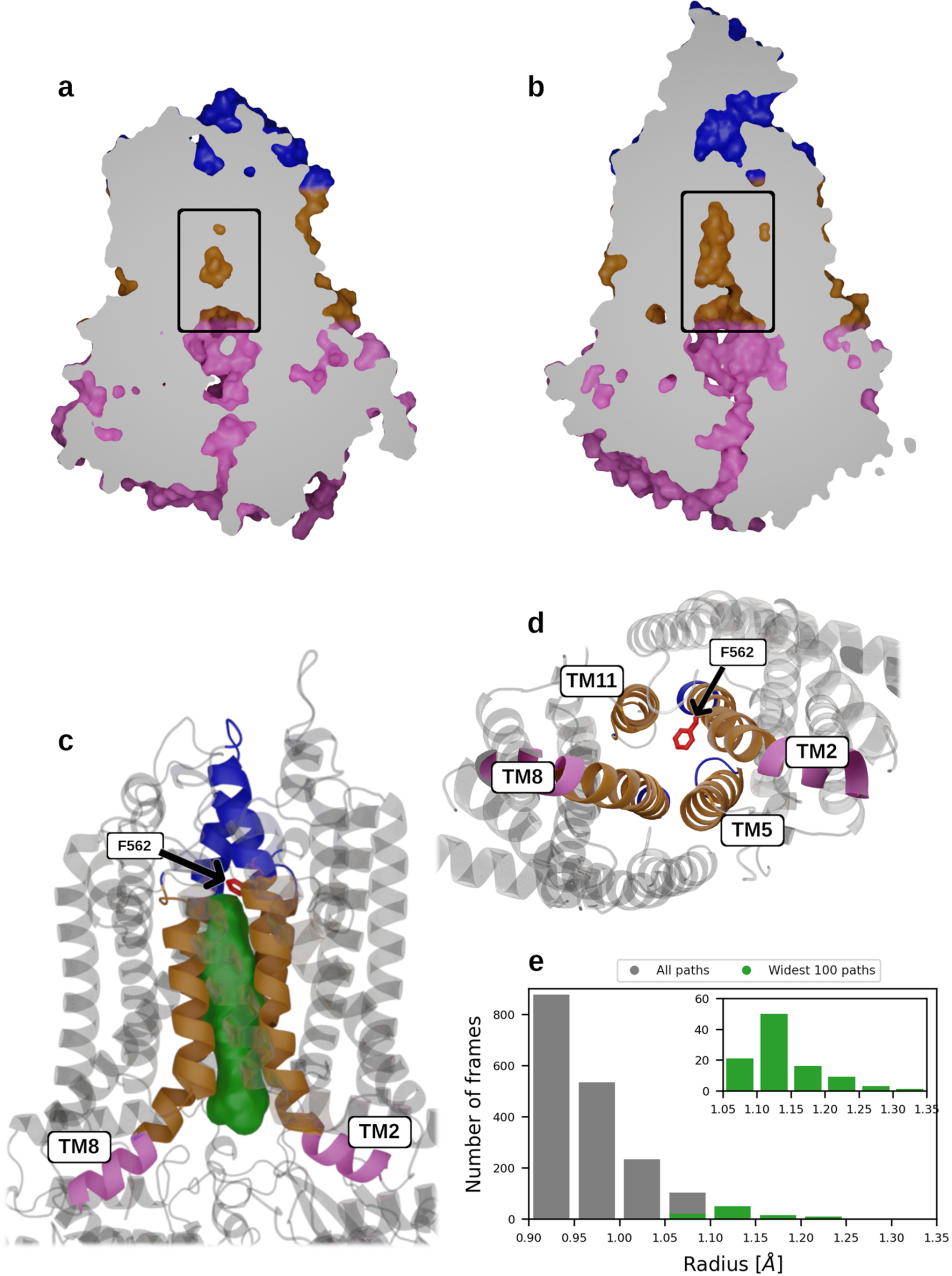
Structure and dynamics of the central cavity and its access path in MtABCG46. **a** Sliced surface side view of the MtABCG46 model obtained from AlphaFold2 featuring an occluded central cavity (indicated by a black rectangle). **b** Sliced surface side view of a cryo-EM structure of ScPDR5 (PDB ID: 7p04) with an open cavity (indicated by a black rectangle). **c** Structural representation of part of MtABCG46, showing the mutated residue F562 (in red sticks), the overall volume of the access path ensemble (green), and TMD helices forming it (intracellular region in pink, transmembrane region in gold, and extracellular region in blue). **d** The bottom view on transmembrane region of MtABCG46 from intracellular side, showing mutated residues F562 (in red sticks), and four TMD helices forming the path and central cavity (intracellular region in pink and transmembrane region in gold). **e** Bottleneck radius distribution of access path ensemble connecting the cavity in MD simulations of MtABCG46, the widest 100 paths are colored green and shown in the inset

The MtABCG46 model obtained from AlphaFold2 has good overall quality according to the pLDDT score [31], with limited confidence for only a few regions (Supplementary Fig. 2a). Moreover, these regions with the low pLDDT scores correspond to highly dynamic motifs in related ABCG proteins [26, 28, 29], for which single structures are hard to define. According to the template modeling score (TM-score) [51], the model has a very similar fold to the recent full-size PDR5 structure of *Saccharomyces cerevisiae* (ScPDR5) [28], human ABCG1 (HsABCG1) [26], and half-size human ABCG2 (HsABCG2) [29] with TM-scores of 0.82, 0.72, and 0.66, respectively (Supplementary Fig. 3). In the structure, the typical arrangement of domains in a full-size ABCG transporter is clearly visible (Supplementary Fig. 2b), with TMD regions, each composed of 6 α-helices, and the two half-size domains joined by a ~ 55-residue linker region. A comparison of central cavities of MtABCG46 and ScPDR5 revealed a small disconnected cavity in the TMD region of MtABCG46 (Fig. 1a). In contrast, a wide-open cavity in the TMD region that directly connects with the cytosolic environment was observed in ScPDR5 (Fig. 1b).

To explore possible paths enabling access of cognate ligands into the central cavity, we analyzed molecular dynamics (MD) simulations for temporarily opened continuous internal voids formed within the MtABCG46 structure using CAVER 3.0 [68] and TransportTools [69]. This revealed a network of putative transport paths from the bottom of the cavity to the intracellular, extracellular, and membrane regions (Supplementary Fig. 4a). To help identification of the likeliest localization of a functionally relevant entrance to such a path along the membrane’s *Z*-axis, we ran six independent MD simulations of systems composed of the endogenous MtABCG46 substrate, liquiritigenin, and a membrane. These simulations showed that liquiritigenin preferentially stayed between the heads and tails of the membrane phospholipids (Supplementary Fig. 4b, c). Consequently, among all paths that opened in this region, the most prevalent (open for ~ 9% of the total simulation time) was the third-ranked one (Supplementary Table 3) with an average bottleneck radius of 0.97 ± 0.06 Å (Fig. 1e). This path provided the most straightforward access to the central cavity from the intracellular region (Fig. 1c and Supplementary Fig. 4d). Interestingly, an equivalent path was practically undetectable in simulations of MtABCG46 without bound ATP molecules (Supplementary Fig. 5). The remaining paths leading to the intracellular region were not considered due to their much lower frequency and much longer, curved geometry (Supplementary Table 4).

### Phylogenetic analyses of residues of the MtABCG46 central cavity

**Fig. 2:**
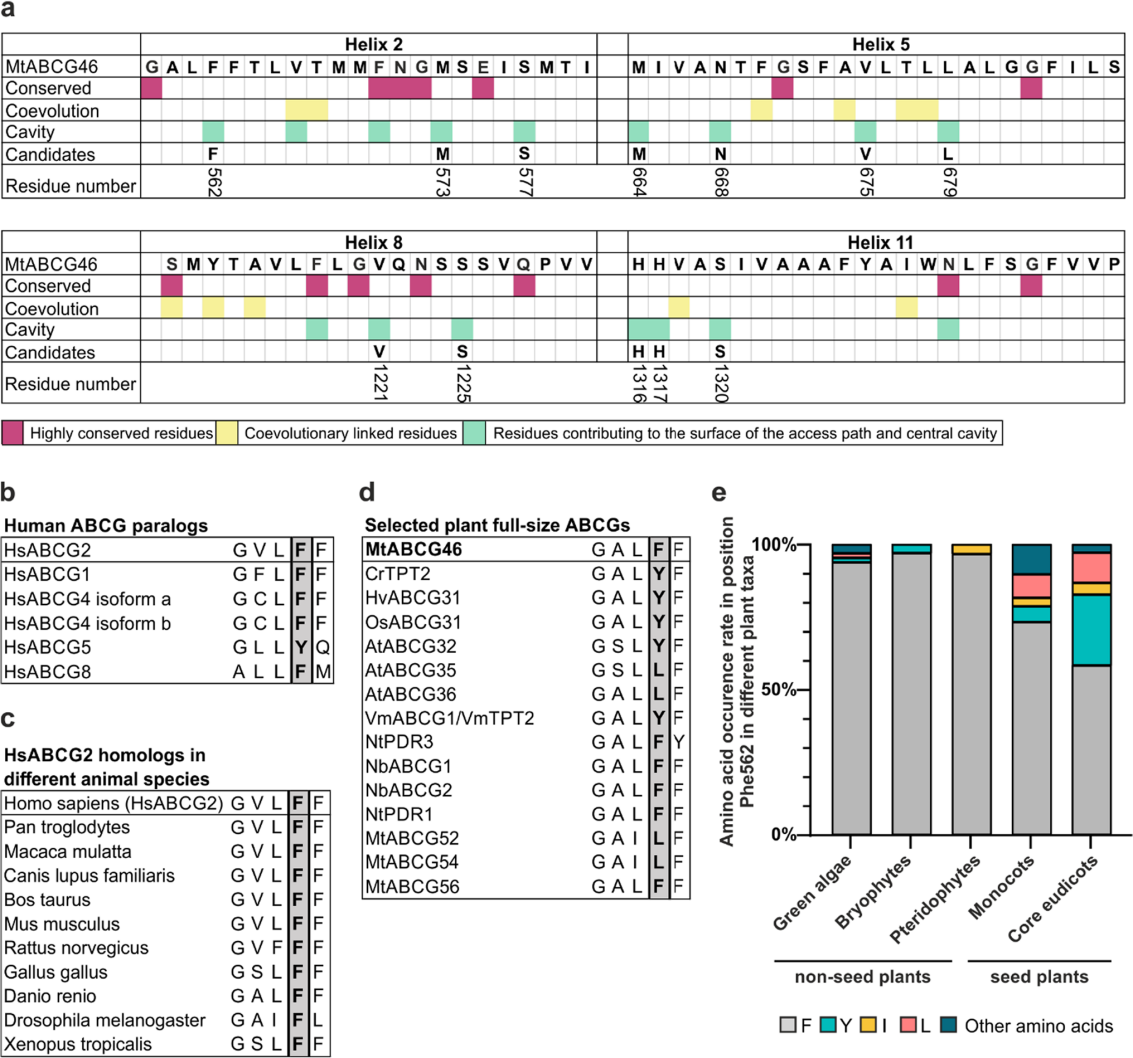
Residue selection for the site-directed MtABCG46 mutagenesis. **a** Schematic representation of candidate residues in sequences of MtABCG46 transmembrane helices 2, 5, 8, and 11 selected using conservation degrees (with Consurf), co-evolutionary parameters (with Gremlin), and the structural data. Candidate residues numbered in accordance with the MtABCG46 sequence. Alignment of the region corresponding to surroundings of F431 in HsABCG2 for human ABCGs (**b**) and animal homologs (**c**). **d** Alignment of the region corresponding to surroundings of F562 in MtABCG46 for selected plant full-size ABCG transporters. **e** Frequencies of occurrence of indicated amino acids in the residue corresponding to MtABCG46 F562 in full-size ABCG sequences of indicated taxa

To investigate the importance of the architecture of internal voids in MtABCG46 for the passage of specific phenylpropanoids, we mapped residues contributing to the surface of the access path and central cavity during the MD simulations (Supplementary Fig. 6). We hypothesized that variations in the recognition and transport of diverse molecules by various ABCG proteins may arise from differences in the amino acid sequences that form the cavity. Based on this assumption, we generated multiple sequence alignment (MSA) of predicted amino acid sequences of 1839 plant full-size ABCG transporters extracted from the 1KP project [42, 43]. The latter collected transcriptomic data from more than 1000 species spanning a diversity of plant kingdom. The sequences were analyzed using the ConSurf [46, 47] and Gremlin [48, 49] servers. We then selected residues that contribute to the central cavity, are not fully conserved (ConSurf grade ≤ 8), and display variability that could not be readily explained by co-evolutional links with other residues (Fig. 2a).

One of the selected amino acids, residue F562 in TMD helix 2, particularly drew our attention due to its correspondence to F431 in HsABCG2 (Fig. 2 and Supplementary Fig. 7), a putatively important residue for ligand recognition and binding [75]. Residue F431 is highly conserved in human ABCG transporters and fully conserved among ABCG2 homologs in several animal species (Fig. 2b, c). However, in plant full-size ABCG transporters, variations at the F562 position include amino acids such as tyrosine, leucine, and isoleucine (Fig. 2d). Intriguingly phylogenetic analyses revealed that the prevalence of amino acids other than phenylalanine at this position is significantly higher in seed plants than in non-seed plants (Fig. 2e). Notably, such expansion of variability in seed plants was not observed for other residues that directly contribute to the cavity surface (Supplementary Fig. 8). Since seed plants have a higher degree of chemodiversity, emphasized in associated specialized metabolism, this led to the conclusion that variability at this position might be meaningful for plant ABCG transporters and/or possibly MtABCG46 selectivity.

### F562 substitutions profoundly affect the selectivity of MtABCG46-mediated transport

To address the importance of F562 for MtABCG46-mediated transport of phenylpropanoids, we substituted it for the other two most frequent amino acids at this position in plant full-size ABCG transporters, tyrosine and leucine (Fig. 2e), as well as alanine. Alanine is often used in such analyses as it eliminates the sidechain beyond the β carbon, but does not alter the main-chain conformation [76].

**Fig. 3:**
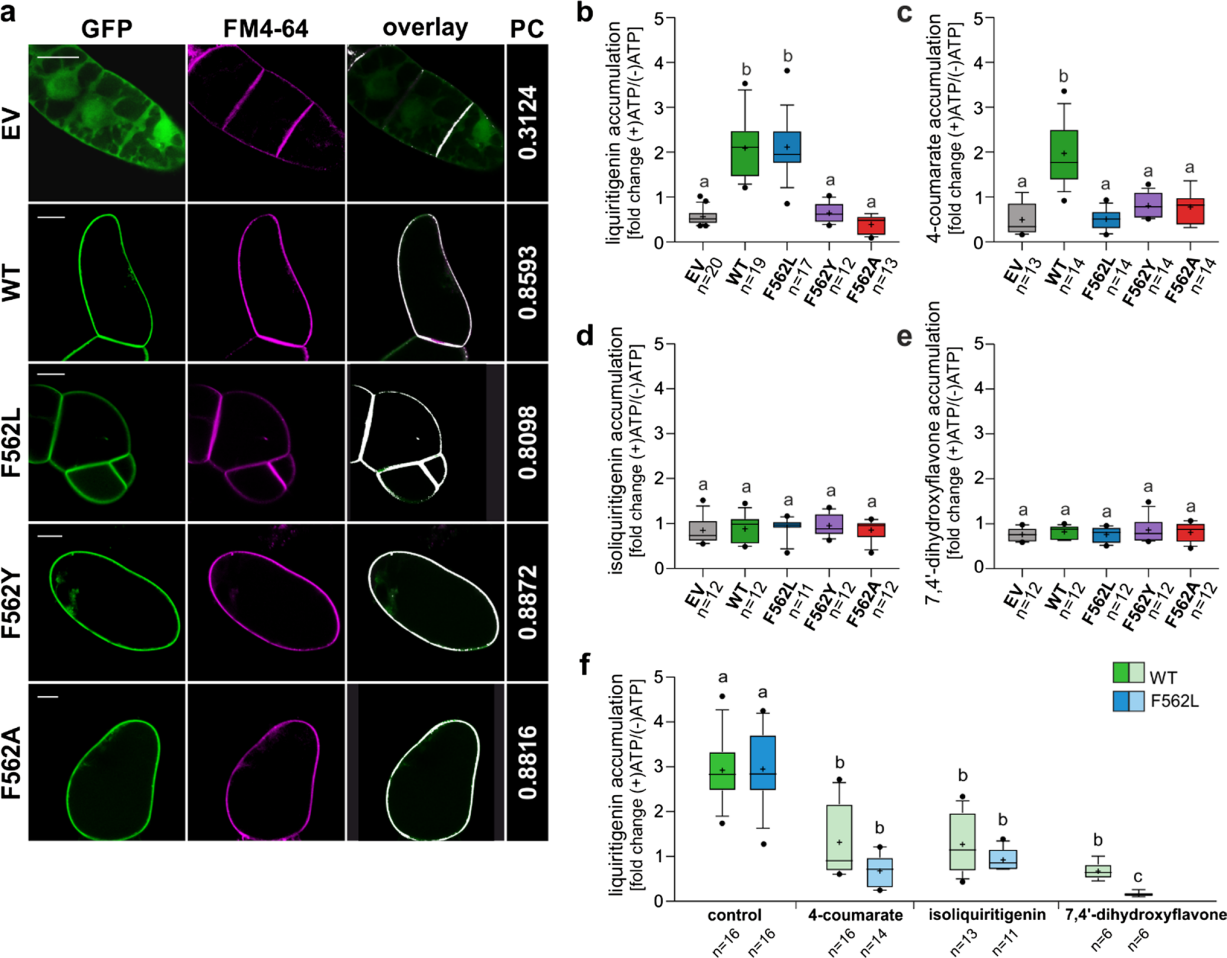
Transport assays with microsomes derived from BY2 suspension cell cultures. **a** Plasma membrane localization of the MtABCG46 variants tagged with GFP: WT, F562L, F562A, and F562Y in BY2 suspension cell cultures. Cell cultures expressing empty vector (EV) were used as a control. Images with GFP and FM4-64 fluorescence pseudo-colored in green and magenta, respectively. PC—Pearson’s correlation coefficients for the colocalization of GFP and FM4-64 in the plasma membrane, visualized as an overlay. Scale bars, 20 µm. Transport of liquiritigenin (**b**), 4-coumarate (**c**), isoliquiritigenin (**d**), and 7,4′-dihydroxyflavone (**e**) in microsomes derived from BY2 suspension cell cultures expressing empty vector (gray) or indicated variants of MtABCG46: WT (green), F562L (blue), F562Y (purple), and F562A (red). **f** Competitive transport of liquiritigenin versus 4-coumarate, isoliquiritigenin or 7,4′-dihydroxyflavone in microsomes expressing WT and F562L MtABCG46, *n* ≥ 6 means ± SD from two to three biological replications, each with at least three technical replications. **b**–**f** Values are presented as fold change between (+)ATP and (−)ATP as a control. In each box-and-whiskers plot: the central black line indicates the median; ‘+’ indicates the mean; the box extends from the 25th to 75th percentile; the whiskers extend from the 10th to the 90th percentile, and points below and above the whiskers are marked by individual dots. Different lowercase letters indicate significant differences: *P* < 0.001 (**b**); *P* < 0.005 (**f**); *P* < 0.01 (**c**); *P* < 0.05 (**d**, **e**). *P* values, determined by the Kruskal–Wallis test with a post hoc Dunn’s multiple comparison test (**b–f**) and unpaired *t* test with Welch’s correction or Mann–Whitney test (**f**) can be found in Supplementary Statistical Data

All our MtABCG46 variants, including the native form (hereafter: wild type, WT), tagged with GFP at the N terminus, were introduced into BY2 tobacco suspension cell cultures, a well-established heterologous expression systems for biochemical studies of ABCG proteins [8, 77]. To confirm the presence and correct localization in the plasma membrane (PM) of the transporters in BY2 lines, the colocalization of GFP-tagged MtABCG46 variants with a PM marker, FM4-64, was checked with confocal microscopy (Fig. 3a).

Effects of mutations were investigated by ATP-dependent, MtABCG46-mediated, transport assays of liquiritigenin, 4-coumarate, isoliquiritigenin, and 7,4′-dihydroxyflavone into PM inside-out vesicles derived from BY2 lines. Consistent with previous observations [24], liquiritigenin and 4-coumarate accumulated in vesicles from lines expressing WT MtABCG46 (Fig. 3b, c) but not isoliquiritigenin or 7,4′-dihydroxyflavone (Fig. 3d, e). Further experiments with lines expressing variants of MtABCG46 revealed that F562Y and F562A substitutions abolished transport of liquiritigenin and 4-coumarate (Fig. 3b, c). None of the variants were able to translocate isoliquiritigenin nor 7,4′-dihydroxyflavone (Fig. 3d, e). Interestingly, the F562L variant, accumulated liquiritigenin similarly to the WT MtABCG46, however, it was not able to transport 4-coumarate (Fig. 3b, c).

Previous research has shown that liquiritigenin is a competitor of 4-coumarate in MtABCG46-mediated transport [24]. Analyses of liquiritigenin transport in the presence of 4-coumarate confirmed that this effect is mutual, i.e., 4-coumarate also reduces the rate of MtABCG46-dependent liquiritigenin transport (Fig. 3f). Experiments also revealed that although F562L cannot transport 4-coumarate, the latter is still a competitor for liquiritigenin (Fig. 3f). Interestingly, presence of non-transported compounds isoliquiritigenin and 7,4′-dihydroxyflavone also negatively affected the MtABCG46-mediated transport of liquiritigenin. Moreover, this susceptibility was increased in F562L variant compared to the WT, what was significantly visible in case of 7,4′-dihydroxyflavone (Fig. 3f).

### F562 substitutions affect the viability of the transient access path through rearranged TMD helices

In MD simulations of our four variants (WT, F562L, F562Y, and F562A), there were no significant differences in overall stability or flexibility between the mutant and WT proteins (Supplementary Figs. 9–12). Structurally, the introduced modifications of residue 562 disrupted non-covalent contacts (Supplementary Fig. 13), changing the bending and twisting angles of the TMD helices forming the access path (Supplementary Figs. 14, 15). In WT, residue F562 maintained a parallel displaced π-stacking interaction with residue F684 during the simulations (Supplementary Fig. 13a), a feature shared with the F562Y mutant (Supplementary Fig. 13c). Loss of this non-covalent contact in F562A resulted in a considerable displacement of TMD helix 5 (Supplementary Fig. 13d). F562L also lost the π-stacking interaction but maintained stability of this helix (Supplementary Fig. 13b) with interhelical space maintained at the level of WT (Supplementary Fig. 16a, b), likely due to remaining Van der Waals interactions of its sidechain. Interestingly, the displacement of TMD helix 5 in F562A allowed marked bending and to some extent twisting of TMD helix 8 (Supplementary Figs. 14c, 15c). Furthermore, the bending of TMD helices markedly reduced the available space between the helices and resulting in a more packed structure. This reduction of available space was observed in both F562A and F562Y mutants (Supplementary Fig. 16c, d), although no significant bending of TMD helix 8 was observed in the F562Y mutant. In this case, the additionally introduced hydroxyl group in F562Y formed an H-bond with Y1213 in TMD helix 8 during the whole simulation time. This pulled the modified residue and Y1213 closer to each other, thereby rearranging the orientation of the neighboring residues in TMD helix 8 and changing its twist helical angle (Supplementary Fig. 15c). Moreover, residue Y1213 formed an H-bond with N1331, a highly conserved residue (Fig. 2a), in all performed MD simulations. Consequently, disturbance of the normal behavior of these residues could influence ligands’ binding in the central cavity. To explore this possibility, we investigated the availability of polar interactions provided by residues of the central cavity. We found only three polar residues around the deepest part of the central cavity: Y1213, T1214, and N1331. Of these, T1214 has limited accessibility as it is oriented outwards of the cavity, and Y1213 maintains constant H-bond contact with the ketone group of N1331 in all MD simulations. Hence, the amine group of N1331 is the only one to act as a hydrogen donor. Notably, a high conservation score was obtained for residue N1331 in our MSA analysis (ConSurf grade 9), indicating that it has functional importance. In our MD simulations, all investigated mutations of residue F562 resulted in a considerable change in the conformation of N1331 (Supplementary Fig. 17) perturbing the putative interaction with bound ligands.

**Fig. 4:**
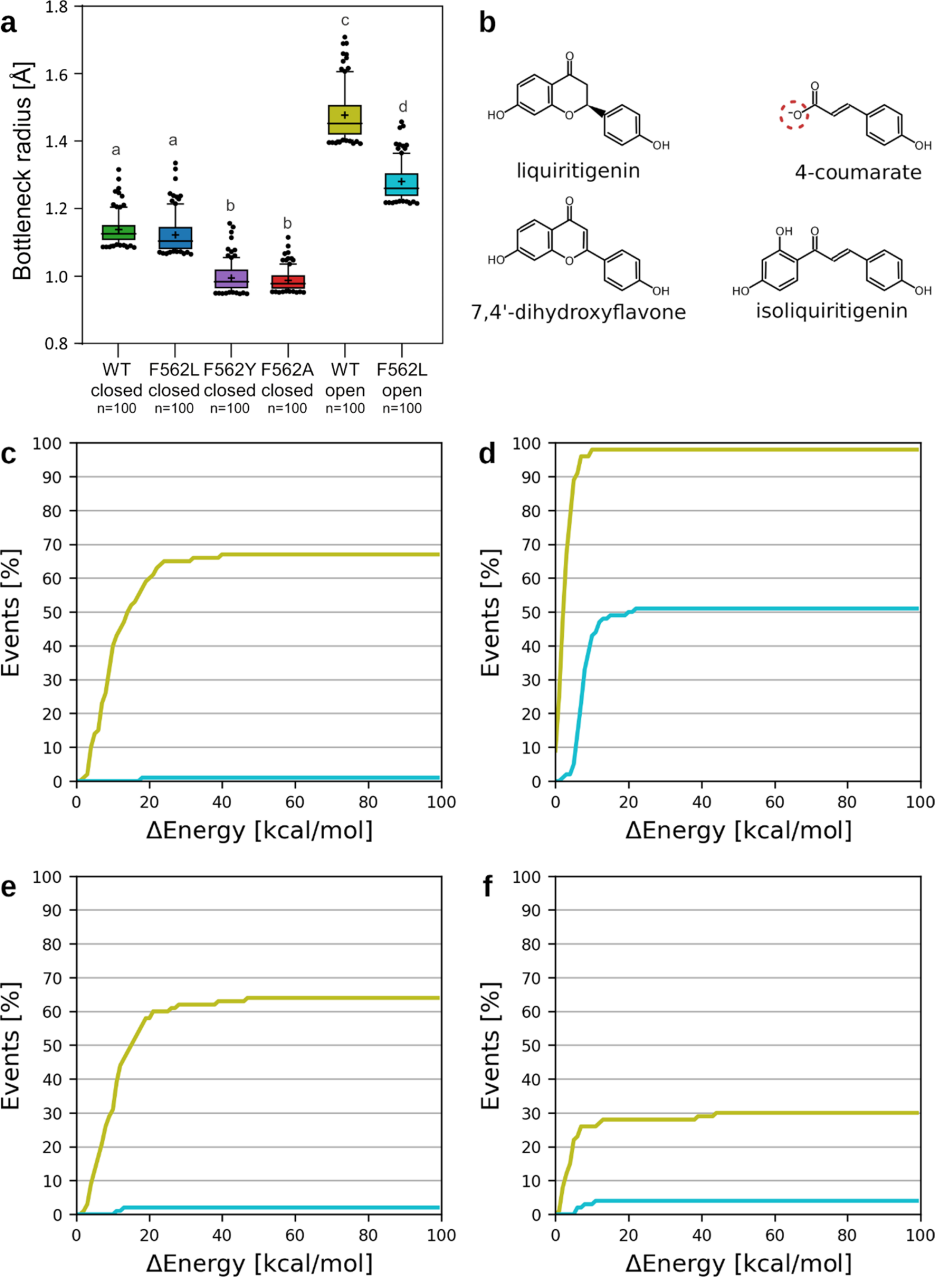
Effects of F562 mutations on the accessibility of the path to the central cavity of MtABCG46. **a** Radius of the probe fitting the bottleneck region of the 100 most open access paths in MtABCG46. In each box-and-whiskers plot: the center black line indicates the median; ‘+’ indicates the mean; the box extends from the 25th to 75th percentile; the whiskers extend from the 10th to the 90th percentile, points below and above the whiskers are marked as individual dots. Different lowercase letters indicate significant differences, *P* < 0.0005. *P* values, determined by the Kruskal–Wallis test with a post hoc Dunn’s multiple comparison test, can be found in Supplementary Statistical Data. **b** Structures of ligands evaluated with CaverDock. The atom of 4-coumarate in red circle could be also be present in protonated form. Comparison of the migration for the two protonation forms is available in Supplementary Fig. 22. Cumulative distributions of energetic barriers for migration to the internal cavity for the widest 100 paths for liquiritigenin (**c**), 4-coumarate (**d**), 7,4′-dihydroxyflavone (**e**), and isoliquiritigenin (**f**). In **c–f** curves obtained from experiments with WT, and F562L in IF-open states are marked in pale green and cyan, respectively

Nonetheless, there were considerable differences in the availability of access paths leading to the central cavity, which were lower in all mutants than in the WT (Supplementary Table 3 and Supplementary Fig. 18a). The 100 widest paths were almost as wide in the F562L mutant as in WT, but they were markedly narrower in F562Y and F562A mutants (Supplementary Fig. 18b), mainly because they had more constricted entrances (Fig. 4a, Supplementary Figs. 16, 19), presumably hampering access of bulky molecules to the central cavity. Accordingly, we hypothesized that F562Y and F562A mutations affect the structural arrangement of residues forming the access path, making it less permissive for migration of ligands and thereby significantly reducing its overall transport capability.

To address this possibility, we assessed the viability of the 100 widest paths in each variant for the access of liquiritigenin and 4-coumarate, as well as isoliquiritigenin and 7,4′-dihydroxyflavone, which are chemically similar but not effectively transported by MtABCG46 (Fig. 4b), from the intracellular environment into the central cavity using CaverDock [70]. Each run of CaverDock yields an energetic profile of the binding energy between a ligand and protein along the path, which can be translated as an energetic barrier that must be overcome for a ligand to reach the central cavity (Supplementary Fig. 20). Such an approach has proven utility in detecting hotspot residues for protein engineering [78], and correlating energetic barriers with biochemical rates [79, 80]. The cumulative distribution of the energy barriers for all ligand migration events resulting in favorable binding of ligands into the central cavity showed that none of the ligands except for 4-coumarate could reach the central cavity effectively (Supplementary Fig. 21). Even for WT and F562L, the fraction of successful migration events was surprisingly low (about 20%, see Supplementary Fig. 21), clearly indicating that a more open state of IF conformation is required to efficiently initiate the transport process by ligand migration into the central cavity. Interestingly, the migration efficiency was similar also when the neutral form of this ligand was considered (Supplementary Fig. 22a).

To investigate the putative open state, we have employed umbrella sampling simulations to achieve the partial opening of the transmembrane helices by driving the system toward the conformation observed in the most open state of ScPDR5 as the only viable template for full-size ABCG transporters. Here, we would like to point out that the available ScPDR5 structures feature only limited openings of their central cavity in different IF conformations despite their pleiotropic nature [28] unlike widely open states observed in half-size human ABCGs, which are among others lacking the covalent linker present in full-size ABCGs. Notably, the opening processes in the slowest-driven US simulations were found to have 1.2, 9.4, and 11.6 kcal mol^−1^ higher energetic costs in F562L, F562Y, and F562A, respectively, when compared to WT (Supplementary Fig. 23) at the conformation ensemble most similar to the arrangements observed in the target ScPDR5 (RMSD of ~ 1.5 Å) (Supplementary Fig. 24). This observation suggested that the compact interhelical space formed due to F562A and F562Y mutations (Supplementary Fig. 16c, d) rendered these variants unlikely to adopt such an open state in IF conformation and perform their function in contrast to WT and F562L, which were much more prone to undergo this transition.

The subsequent unbiased simulations of WT and F562L starting from their induced IF-open states, showed a partial reversal toward their closed states, reaching an RMSD of ~ 2.2 and 2.4 Å compared to the target ScPDR5, respectively (Supplementary Fig. 25). Despite such relaxations, these simulations still featured a more frequent opening of the continuous access paths for at least 31 and 12% of the total simulation time in WT and F562L, respectively (Supplementary Table 5). The bottlenecks of these paths could be fitted with spherical probes of up to 1.7 and 1.4 Å radii in WT and F562L, respectively (Fig. 4a and Supplementary Table 5). Notably, the access paths ensemble in the open states of MtABCG46 also included branches that opened laterally to the lipid bilayer (Supplementary Fig. 26), allowing ligands to enter either from the membrane or through the cytosolic region, in contrast to the access paths found in the closed states. Additionally, the interhelical region of WT in the open state underwent significant enlargement along the majority of its length (Supplementary Fig. 16e), expanding not only the entry filter but also the volume of the central cavity. In contrast, conformations from F562L simulations mostly showed only minor enlargements in this region (Supplementary Fig. 16f).

When considering the explicit calculation of ligand migration, performed with CaverDock, the revealed expansions enabled favorable access of all investigated ligands to the central cavity of MtABCG46 WT, exhibiting distinct preference for 4-coumarate. It was illustrated by almost 100% rate of successful migration events (Fig. 4d), irrespectively of the protonation form (Supplementary Fig. 22b). The much bulkier liquiritigenin and 7,4′-dihydroxyflavone reached the cavity in about 60% of migration events, with a slight preference for the former (Fig. 4c, e). In contrast, the least favorable migration was calculated for isoliquiritigenin, with only 30% rate of successful migration events (Fig. 4f). In the F562L mutant, the preference concerned only the 4-coumarate, which retained about half of its efficiency, compared to WT (Fig. 4d). All bulkier compounds reached similar efficiencies when it comes to migrating to the central cavity of F562L, with much lower success rates and considerably higher energy costs (Fig. 4c, e, f). The partial discrepancy, visible in the overall transport assays, corresponds well with the disruption of the helices and the central cavity, caused, to different degrees, by all three mutations investigated in F562 (Supplementary Fig. 16). This suggests that these mutations may also affect subsequent stages of the transport cycle.

## Discussion

AlphaFold2 has recently proposed suitability for modeling ABCG proteins, with the ability to provide similar levels of accuracy as for soluble proteins [32]. The MtABCG46 structure we obtained using AlphaFold2 has the typical architecture of full-size ABCG transporters described in the literature [81]. Moreover, our MD simulations identified transiently formed access paths to the central cavity from the intracellular region that were narrower than the ones observed in IF-ScPRD5 structures, irrespective of the adopted states (Fig. 1, Supplementary Fig. 27 and Supplementary Tables 4 and 5). Such open cavities were observed in HsABCG1 [26], HsABCG2 [29], and a recently obtained cryo-EM structure, ScPDR5 [29] (Supplementary Fig. 28). Also several substrates such as rhodamine 6G, cholesterol, and mitoxantrone have been found to occupy equivalent regions of ScPDR5, HsABCG1, and HsABCG2, respectively [26, 28, 29]. However, context of the access path to the cavity, as well as its role in substrate recognition and transport, have not been thoroughly investigated.

Tested substitutions of the selected residue F562 in the central cavity (F562L, F562Y, and F562A) severely affected MtABCG46-mediated transport of 4-coumarate and liquiritigenin, highlighting this residue’s importance for phenylpropanoid transport in *M. truncatula*. Transport assays of four phenylpropanoids with MtABCG46 WT showed lack of isoliquiritigenin or 7,4′-dihydroxyflavone transport (Fig. 3d, e), in accordance with indications from CaverDock analyses in IF-open like conformation predicted very good efficiency of migration to the central cavity for both 4-coumarate and liquiritigenin (Fig. 4c, d). In contrast, likelihood of other two compounds to access the central cavity is lower, in particular for metabolic precursor isoliquiritigenin (Fig. 4e, f). We have previously shown that the MtABCG46 promotes response of *Medicago truncatula* to pathogen infection by efficient transport of liquiritigenin but not isoliquiritigenin resulting in the de novo biosynthesis of the pterocarpan phytoalexin medicarpin, derived from the 5-deoxyisoflavonoid branch. Our observation regarding the efficiency of migration to the central cavity supports the potential role of it in this process. This is because, both scenario and pathway wise, such preference toward liquiritigenin but not isoliquiritigenin could be beneficial since the latter is a direct precursor that has to be converted and not transported [23, 24].

Moreover, the observed migration priority is in agreement with the ability of isoliquiritigenin or 7,4′-dihydroxyflavone to interfere with liquiritigenin transport in MtABCG46 WT (Fig. 3f), while lack of clear preference for liquiritigenin entry into the central cavities in F562L mutant (Fig. 4c, e, f) agrees well with the increased susceptibility of this protein to the interference from the same compounds (Fig. 3f). In contrast, the lack of transport capabilities in F562A or F562Y (Fig. 3) is well in line with the observed collapse of the interhelical space lining the access path and consequent overstabilization of IF-closed state caused by their mutations, disallowing the necessary opening for efficient ligand transport (Fig. 4a and Supplementary Fig. 16, 23). Thus, our data indicate that restriction of access to the central cavity is a contributor to substrate selectivity in early stages of the transport process, probably highly relevant for overall activity of the transporter in competitive environment inside the cell, where structurally analogous metabolites are present. Also, the fact that all four phenylpropanoids can still access the central cavity of MtABCG46 WT and F562L clearly reveals the presence of additional molecular mechanisms behind the observed selectivity in subsequent stages of the transport cycle, e.g., recognition of the substrate bound in the central cavity or its stabilization during the subsequent, likely rate-determining, conformational change (from IF to OF) of the transporter.

Similarly to the corresponding residue F431 of HsABCG2, in ABCG46 F562 is located in the direct proximity of the short loop after TMD helix 5 (and TMD helix 11). This loop is called a valve and has been proposed to work as a molecular gate crucial for regulating the conformation-dependent substrate release [29, 33]. A residue in this loop in another full-size plant ABCG transporter from *A. thaliana,* AtABCG36 was also highlighted as potentially important for substrate specificity [18], also supporting our results. Our simulations showed that F562 interacts with at least one of the residues within the valve, namely F684, behavior of which was substantially altered in F562A and F562L variants (Supplementary Fig. 13). This enabled us to speculate, that introduced substitutions of F562 could significantly alter this structural element important for the transport mechanism. The substitutions were also considerably affecting the conformation of highly conserved N1331 (Supplementary Fig. 17), being the only hydrogen donor in the deep part of the central cavity, which could in the consequence affect the substrate binding.

In general, the molecular determinants of sequence changes resulting from protein adaptation to various biochemical needs remain obscure. Our experimental data, together with phylogenetic observations, suggest that adaptation of ABCG proteins associated with evolutionary pressures in plants has resulted in a variability of some key residues, such as F562 (Fig. 2e). Such variability is essential for transporters to fulfill their functional roles in diverse, complex chemical and biological scenarios. We foresee sequence–structure–dynamics exploration fueled by AplhaFold2 presented here as an alternative mean to overcome limitations in structural studies of membrane transporters, which can help to identify residues that define functional properties of that important subfamily of ABC transporters.

## Supplementary Information

Below is the link to the electronic supplementary material.

Supplementary file1 (DOCX 16032 kb)

Supplementary Figures and Tables (PDF) include details on the target region used to obtain the IF-open state of MtABCG46; the MtABCG46 model and comparison with experimental ABC proteins; the localization of access paths leading into central cavity and description of their characteristics; structural and sequential alignment of MtABCG46 and HsABCG2; conservation score of the inner cavity forming residues; RMSD and RMSF of MtABCG46 variants observed in simulations; structural coupling between access path and transmembrane helices; interactions between key residues of transmembrane helices; the description of perturbation of transmembrane helices by mutagenesis; comparison of internal cavities among related ABC transporters; the description of conformational space adopted by N1331 in MtABCG46 variants observed in simulations; duration and restraints applied in individual simulation stages; energetic barriers of ligand transport in MtABCG46 variants in IF open and closed states; potentials of mean force for US simulations for transition from closed to open IF states of MtABCG46 variants. Additionally, the results of statistical analyses are summarized in the Supplementary Statistical Data (XLSX). (XLSX 30 kb)

## Data Availability

Sequence data reported in this article can be found in the NCBI database under the following accession numbers: MtABCG46 (Medtr_2g102670), MtABCG52 (Medtr_8g014360), MtABCG54 (Medtr_5g070320), MtABCG56 (Medtr_2g101090), AtABCG32 (AT2G26910), AtABCG35 (AT1G15210), AtABCG36 (AT1G59870), NbABCG1a (BAR94041), NbABCG2b (BAR94044), NtPDR3 (Q5W274), NtPDR1 (NP_001312599), HvABCG31/EIBI1 (BAK52288), OsABCG31 (Os01g0177900), CrTPT2 (KC511771), VmABCG1/VmTPT2 (KC511773), HsABCG1 isoform X1 XP_011528108, HsABCG2 NP_004818.2, HsABCG4 isoform a NP_001335120.1, HsABCG4 isoform b NP_001335121.1, HsABCG5 NP_071881.1, HsABCG8 NP_071882.1; HsABCG2 homologs: *Homo sapiens* NP_004818.2, *Pan troglodytes* XP_526633.3, *Macaca mulatta* NP_001028091.1, *Canis lupu*s *familiaris* NP_001041486.1, *Bos taurus* NP_001032555.2, *Mus musculus* NP_036050.1, *Rattus norvegicus* NP_852046.1, *Gallus* XP_421638.4, *Danio rerio* NP_001036240.1, *Drosophila melanogaster* NP_001039227.1, *Xenopus tropicalis* NP_476787.1. The sequences from 1KP analysis, predicted AlphaFold2 models, parameters and input files for MD simulations of all MtABCG46 variants, as well as key restart files, results of tunnel geometry and ligand migration analyses are available at https://doi.org/10.5281/zenodo.7002738.
