# Supplementary material for: Restriction of access to the central cavity is a major contributor to substrate selectivity in plant ABCG transporters
Source: Cell Mol Life Sci. 2023 Mar 23;80(4):105. doi: 10.1007/s00018-023-04751-6 (PMC10036432; doi:10.1007/s00018-023-04751-6)
Supplement: Supplementary file 1 — Supplementary file1 (DOCX 16032 kb) [file 18_2023_4751_MOESM1_ESM.docx]

**SUPPLEMENTARY INFORMATION**

**Restriction of access to the central cavity is a major contributor to substrate selectivity in plant ABCG transporters**

**Konrad Pakuła^1,2*^, Carlos Sequeiros-Borja^3,4*^, Wanda Biała-Leonhard^1*^, Aleksandra Pawela^1^, Joanna Banasiak^1^, Aurélien Bailly^5^, Marcin Radom^6,7^, Markus Geisler^8^, Jan Brezovsky^3,4✉^, Michał Jasiński^1,9^ ^✉^**

1. Department of Plant Molecular Physiology, Institute of Bioorganic Chemistry, Polish Academy of Sciences, Poznan, Poland
2. NanoBioMedical Centre, Adam Mickiewicz University, Poznan, Poland
3. Laboratory of Biomolecular Interactions and Transport, Department of Gene Expression, Institute of Molecular Biology and Biotechnology, Faculty of Biology, Adam Mickiewicz University, Poznan, Poland
4. International Institute of Molecular and Cell Biology, Warsaw, Poland
5. Department of Plant and Microbial Biology, University of Zurich, Zurich, Switzerland
6. Department of Structural Bioinformatics, Institute of Bioorganic Chemistry, Polish Academy of Sciences, Poznan, Poland
7. Institute of Computing Science, Poznan University of Technology, Poznan, Poland
8. Department of Biology, University of Fribourg, Fribourg, Switzerland
9. Department of Biochemistry and Biotechnology, Poznan University of Life Sciences, Poznan, Poland

* These authors contributed equally to the work

✉ For correspondence: **Michał Jasiński**, Department of Plant Molecular Physiology, Institute of Bioorganic Chemistry, Polish Academy of Sciences, Z. Noskowskiego 12/14, 61-704 Poznan, Poland (+48) 61 852 85 03 (e-mail [jasinski@ibch.poznan.pl](mailto:jasinski@ibch.poznan.pl)); **Jan Brezovsky**, Laboratory of Biomolecular Interactions and Transport, Department of Gene Expression, Institute of Molecular Biology and Biotechnology, Faculty of Biology, Adam Mickiewicz University, Uniwersytetu Poznanskiego 6, 61-614 Poznan, Poland, and International Institute of Molecular and Cell Biology in Warsaw, 02-109 Warsaw, Poland (+48) 61 829 5839 (e-mail [janbre@amu.edu.pl](mailto:janbre@amu.edu.pl), [jbrezovsky@iimcb.gov.pl](mailto:jbrezovsky@iimcb.gov.pl)).


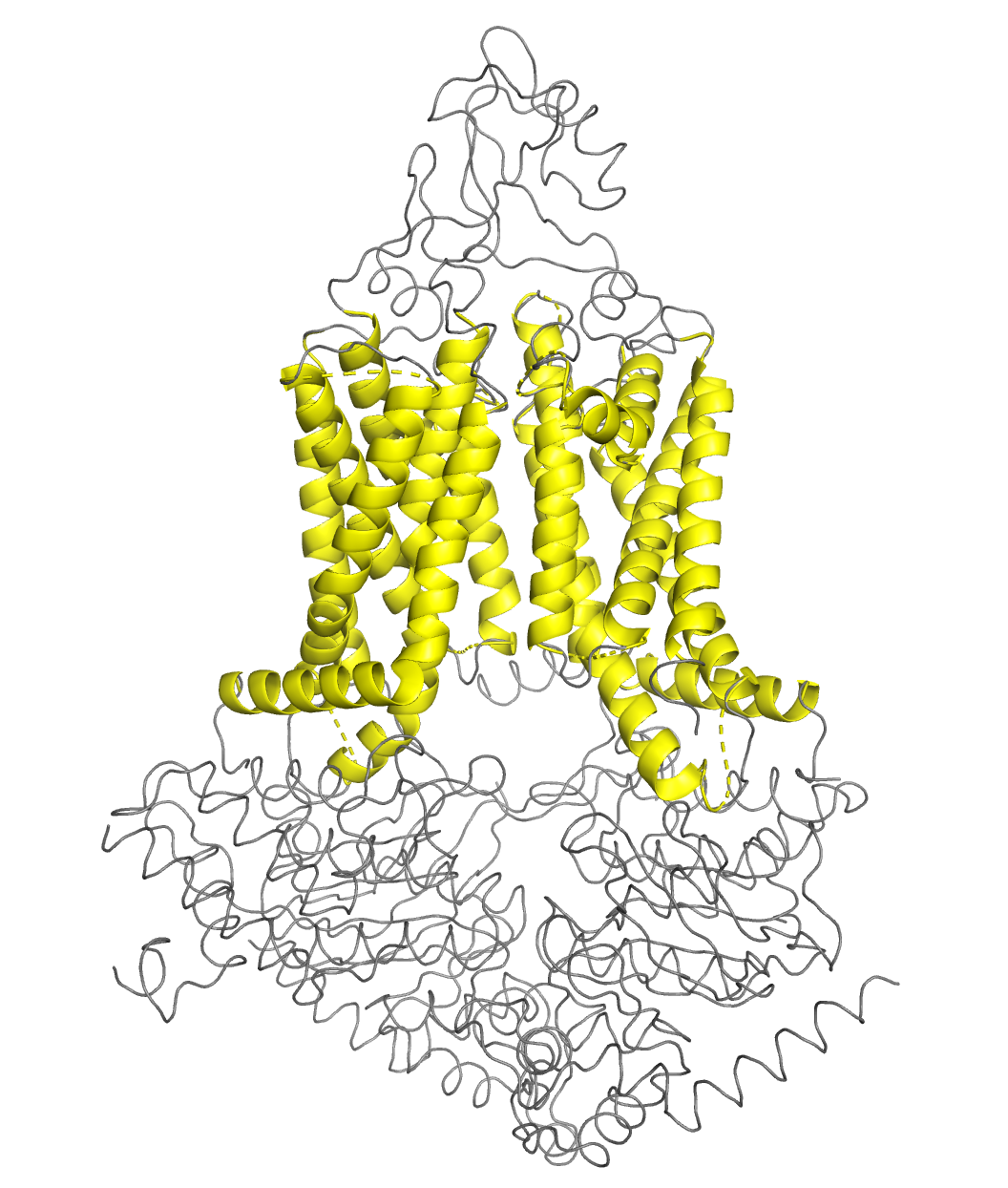


**Supplementary Figure 1: TMD helices of ScPDR5 employed as umbrella sampling target.** Protein structure of ScPDR5 in the conformation bound to rhodamine 6G (PDB ID: 7p05) employed as target for US simulations. Only the structure of the protein is presented for simplicity, with the main chain as gray ribbon, and the TMD helices used as cartoon in yellow.


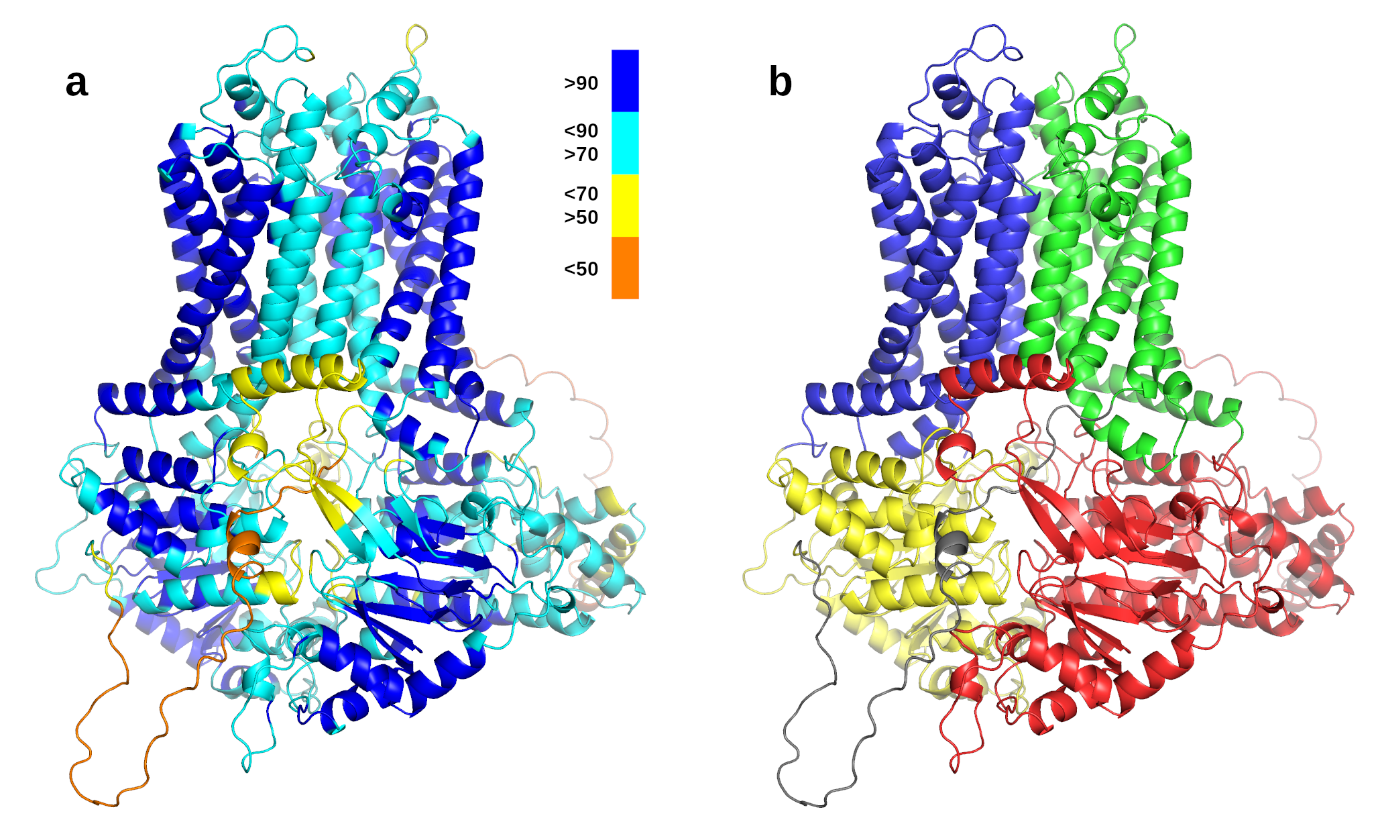


**Supplementary Figure 2: AlphaFold2 model of MtABCG46.** Protein structure of MtABCG46 colored by: a) pLDDT score, and b) subdomains, with NBD1 in red, TMD1 in green, linker region in gray, NBD2 in yellow, and TMD2 in blue.


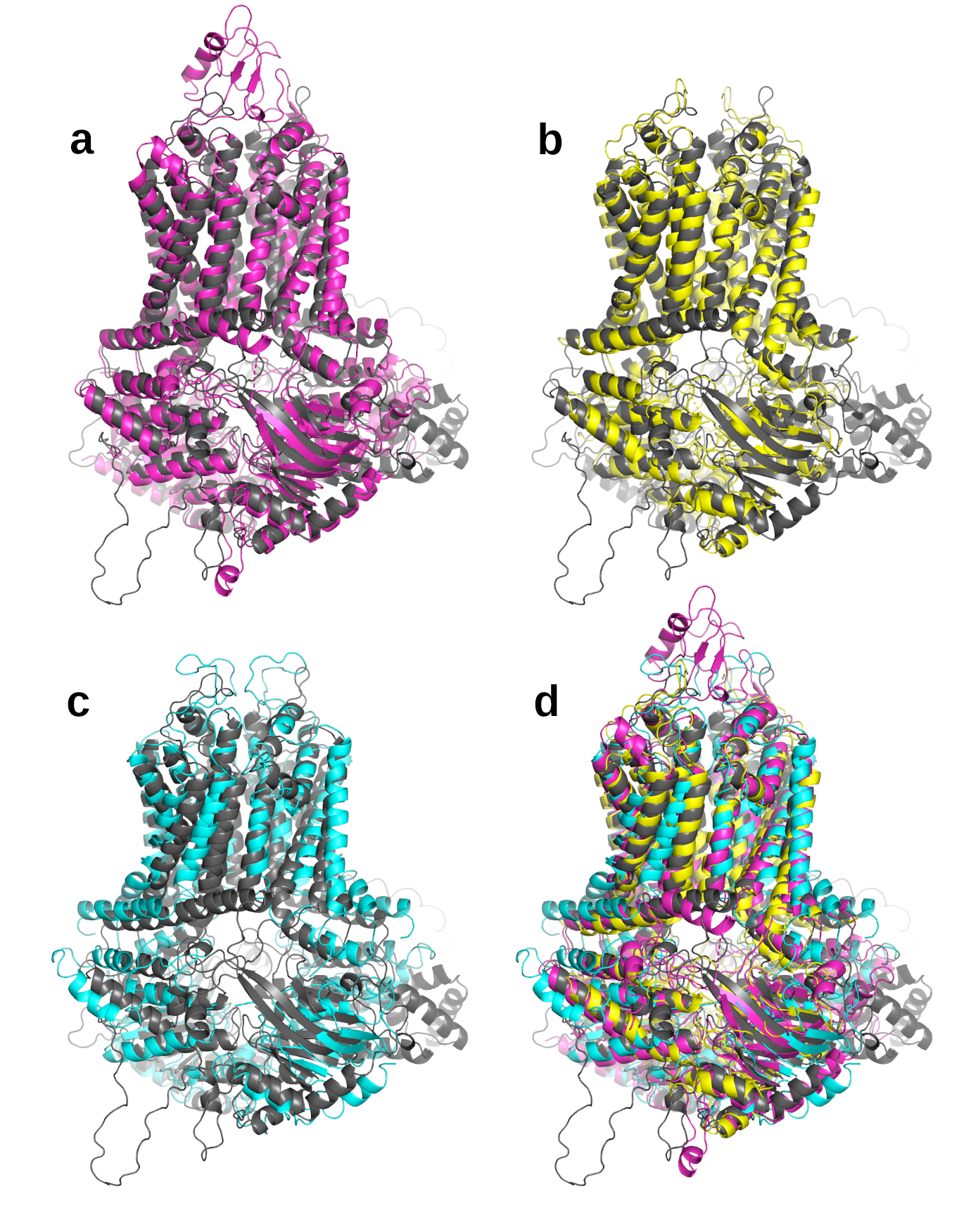


**Supplementary Figure 3:** **Structural alignment of MtABCG46 related ABCG transporters.** **a-d** Structural alignment to MtABCG46 in gray obtained with TM-score for ABCG related proteins: ScPDR5 in magenta (PDB id: 7p04) (**a**); HsABCG1 in yellow (PDB id: 7r8e) (**b**); HsABCG2 in cyan (PDB id: 6vxh) (**c**); all four structures aligned show the same fold (**d**).


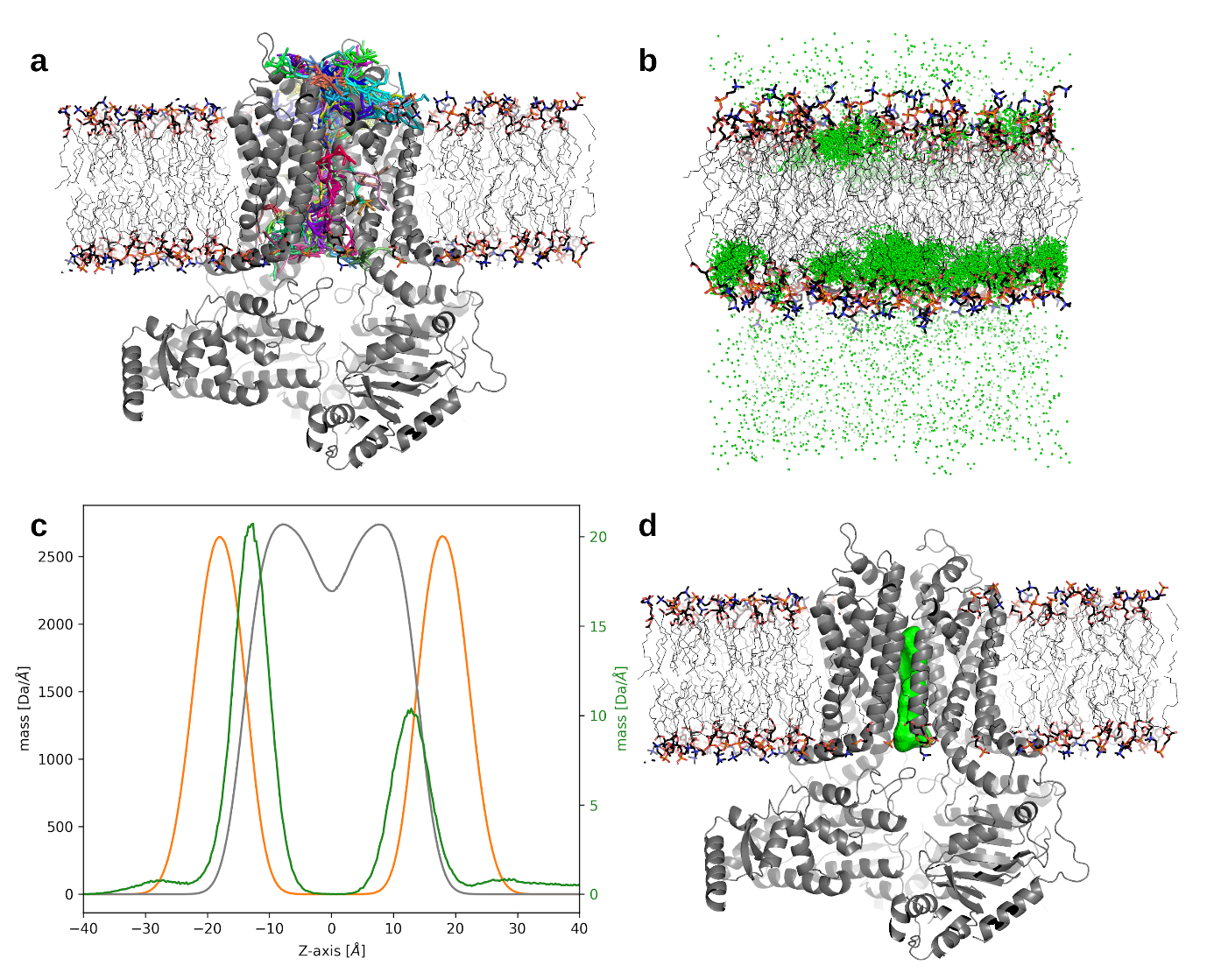


**Supplementary Figure 4:** **Network of access paths in MtABCG46 obtained with TransportTools and liquiritigenin localization.** **a** Clusters of paths (differentially colored sticks) identified in MtABCG46 structure (gray cartoon) embedded in POPC membrane shown with heads as sticks and tails as lines. **b** Preferred liquiritigenin localization in the membrane; each green sphere represents the center of mass of a liquiritigenin molecule per frame. **c** Linear mass density across the Z-axis, POPC heads colored orange and POPC tails as gray with left axis; liquiritigenin colored as green with the right axis. **d** MtABCG46 structure as a gray cartoon with the overall volume of selected access path ensemble represented as a green surface.


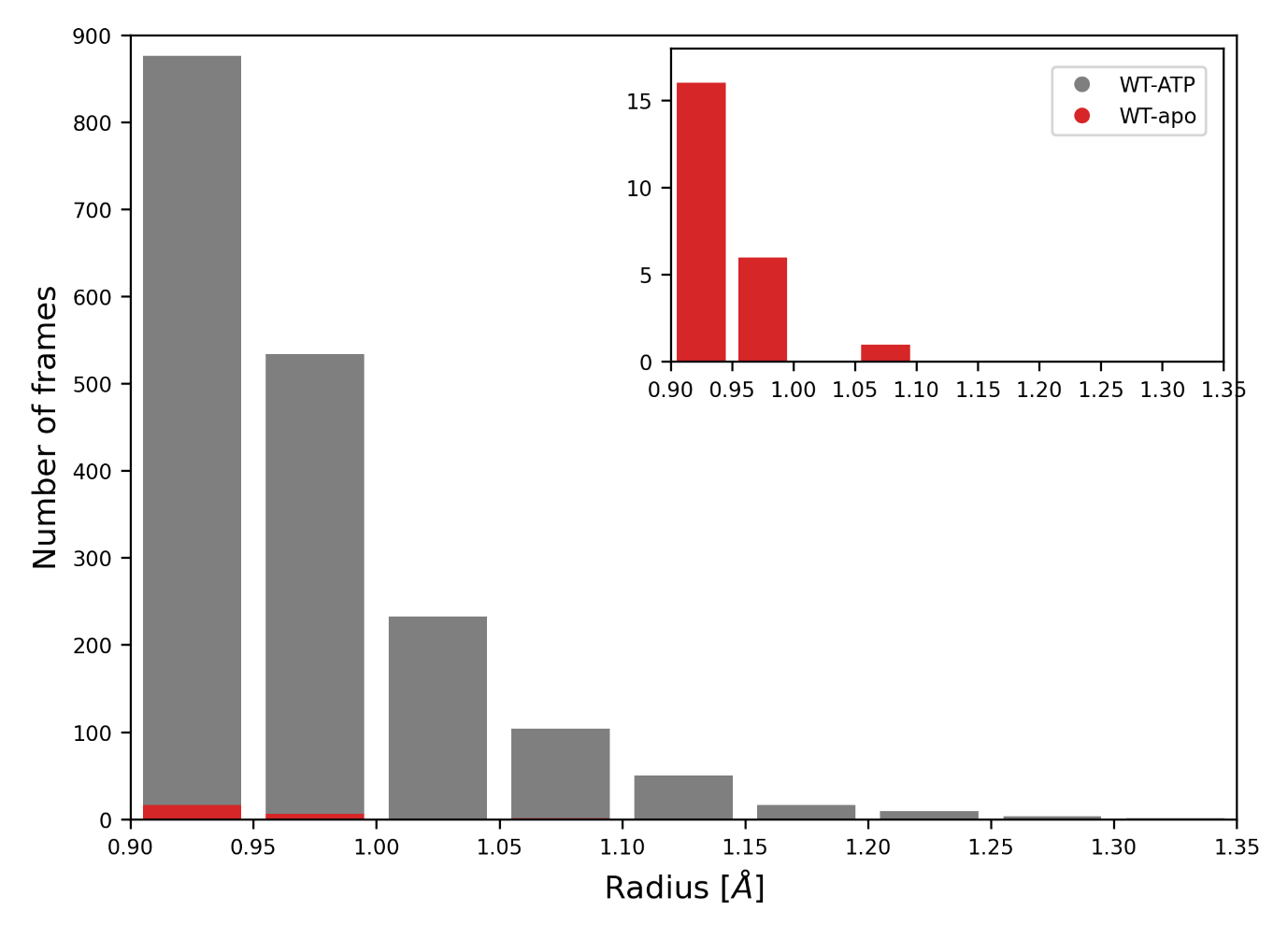


**Supplementary Figure 5: Presence of the access path in MtABCG46 ATP-bound and apo (without ATP and MG^2+^ ions) models.** The amount of frames where the access path was present in the ATP-bound model is colored in gray and in red for the apo form. In the inset, only the results from the apo form are shown.


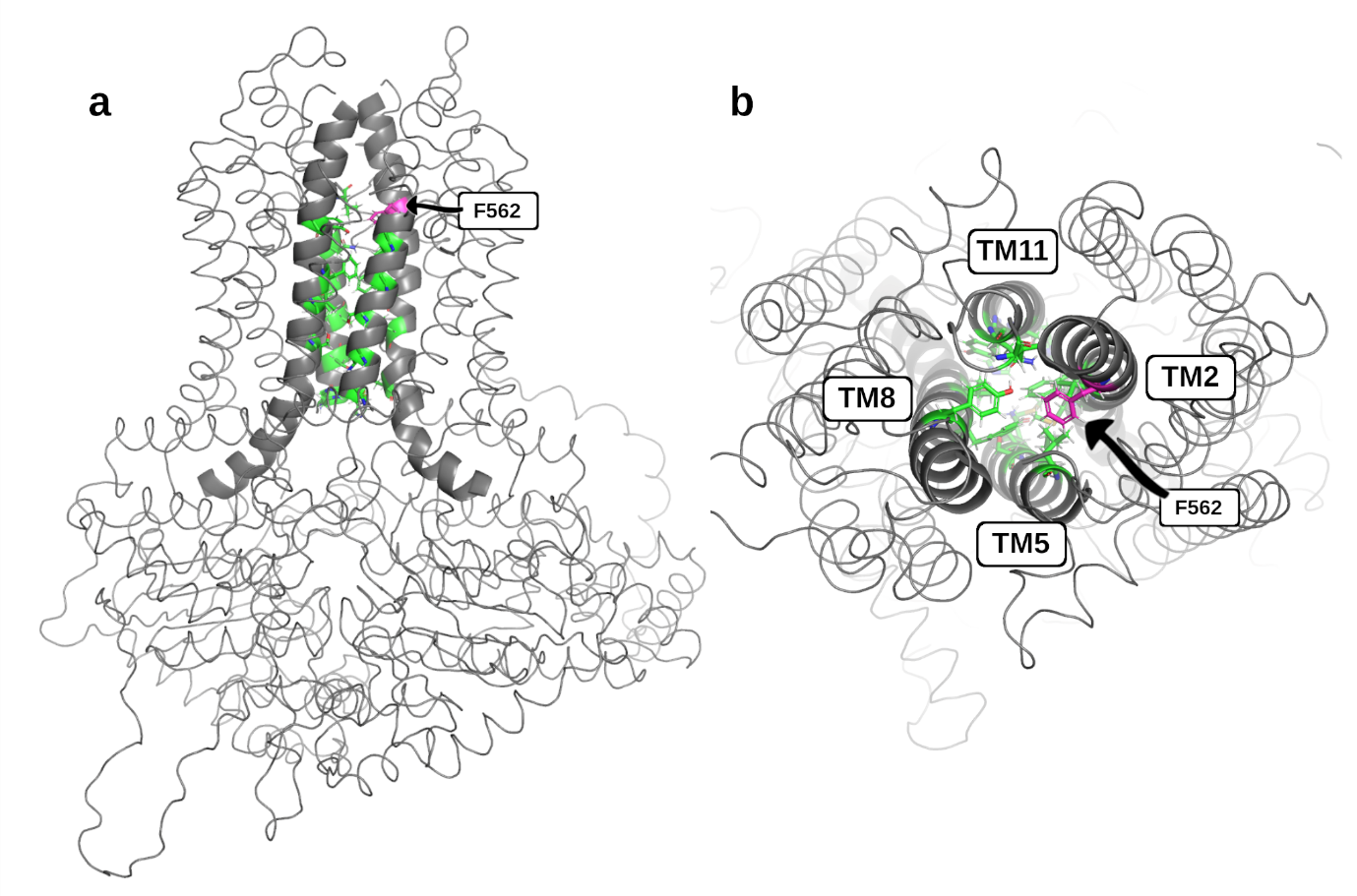


**Supplementary Figure 6: Space between four TMD helices harboring the central cavity and its access path in MtABCG46.** **a** Side view of the model showing the four TMD helices that form the access path in MtABCG46. **b** Top view of lining residues of the access path.


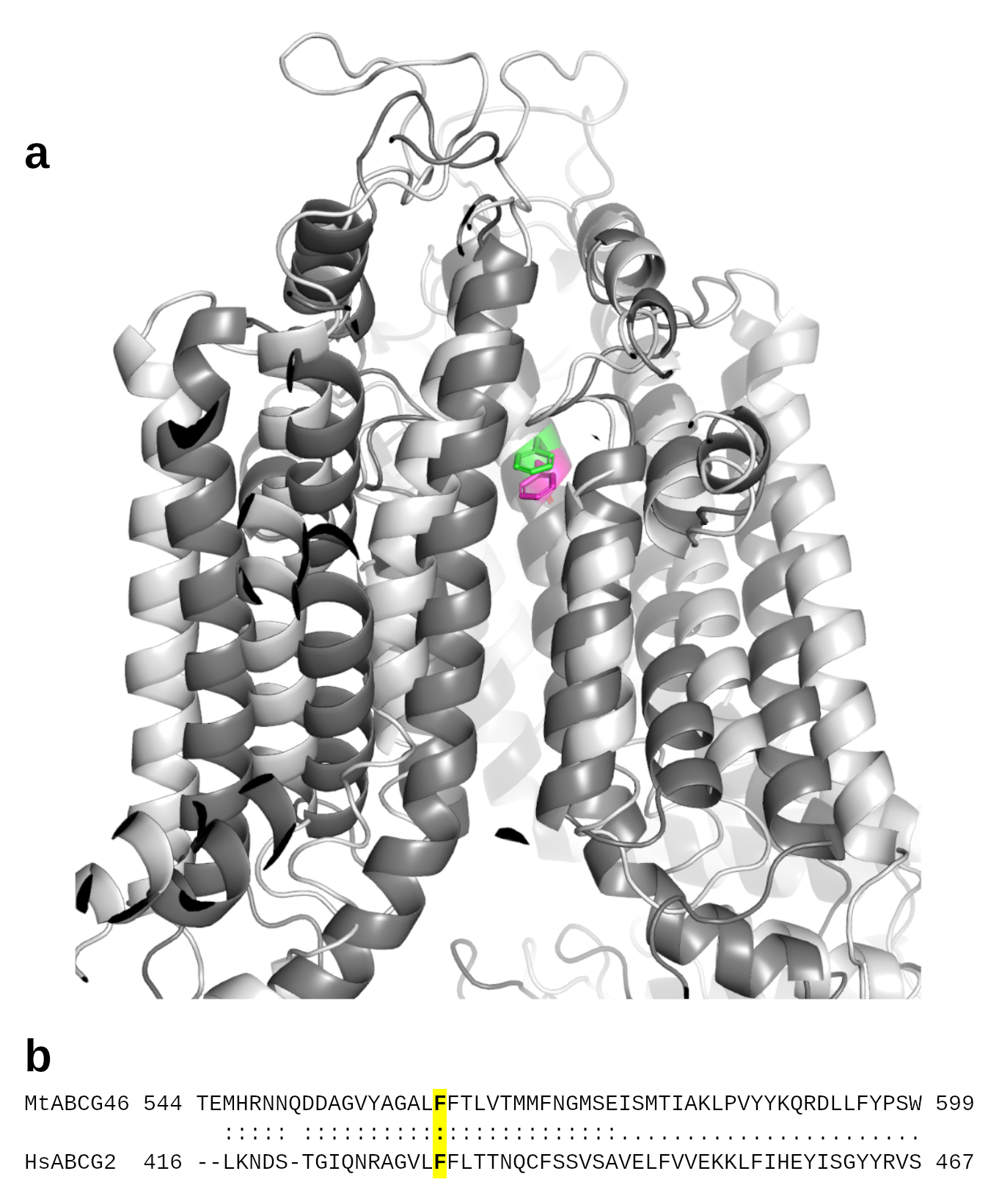


**Supplementary Figure 7: Structural and sequential alignment of MtABCG46 and HsABCG2.** **a** Structure of HsABCG2 in a light gray cartoon with residue F431 highlighted as magenta sticks, and MtABCG46 as a dark gray cartoon with residue highlighted F562 as green sticks. **b** TM-alignment of MtABCG46 and HsABCG2 dimer. F562 of MtABCG46 and corresponding residue of HsABCG2 – F431 marked bold and yellow, where “:” denotes aligned residue pairs of distance < 5 Å, and “.” denotes other aligned residues


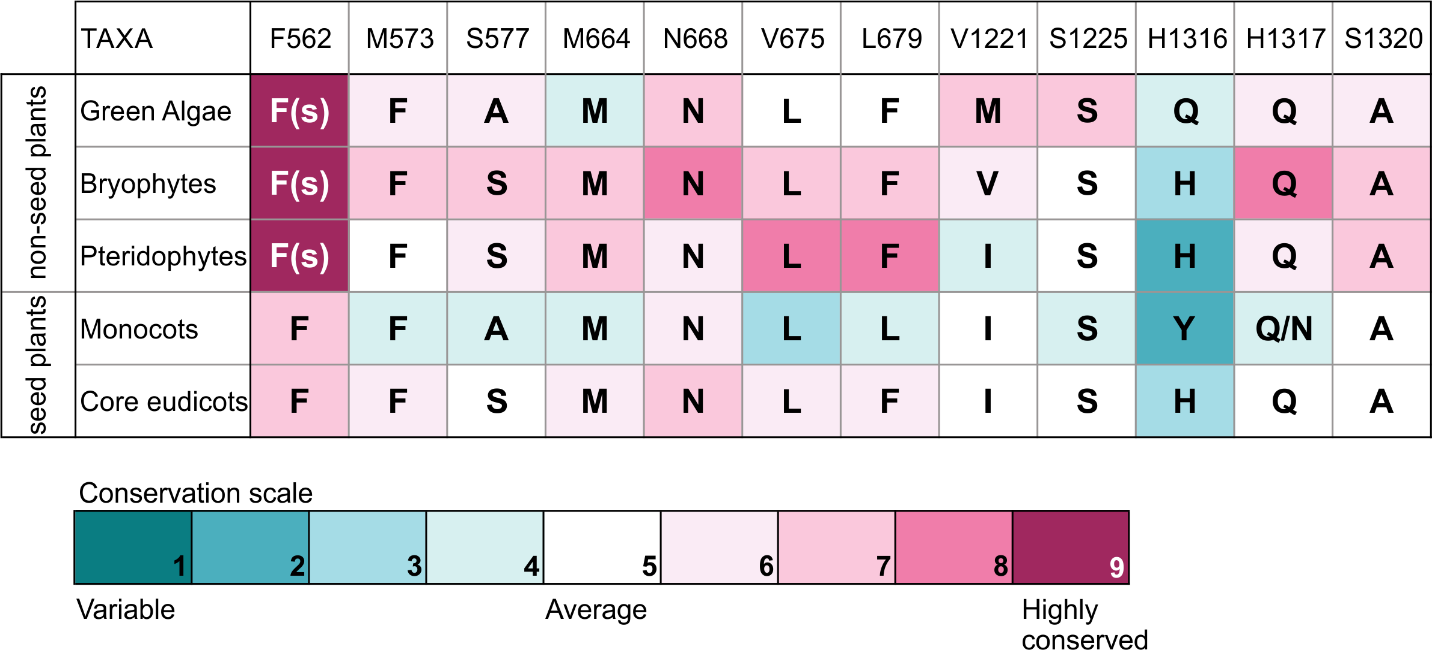


**Supplementary Figure 8: Conservation score of the inner cavity forming residues among different plant taxa based on Consurf analysis.** The most commonly present amino acid in the corresponding residue within taxa is indicated by a single letter amino acid code; (s) corresponds to residues predicted by Consurf as structural.


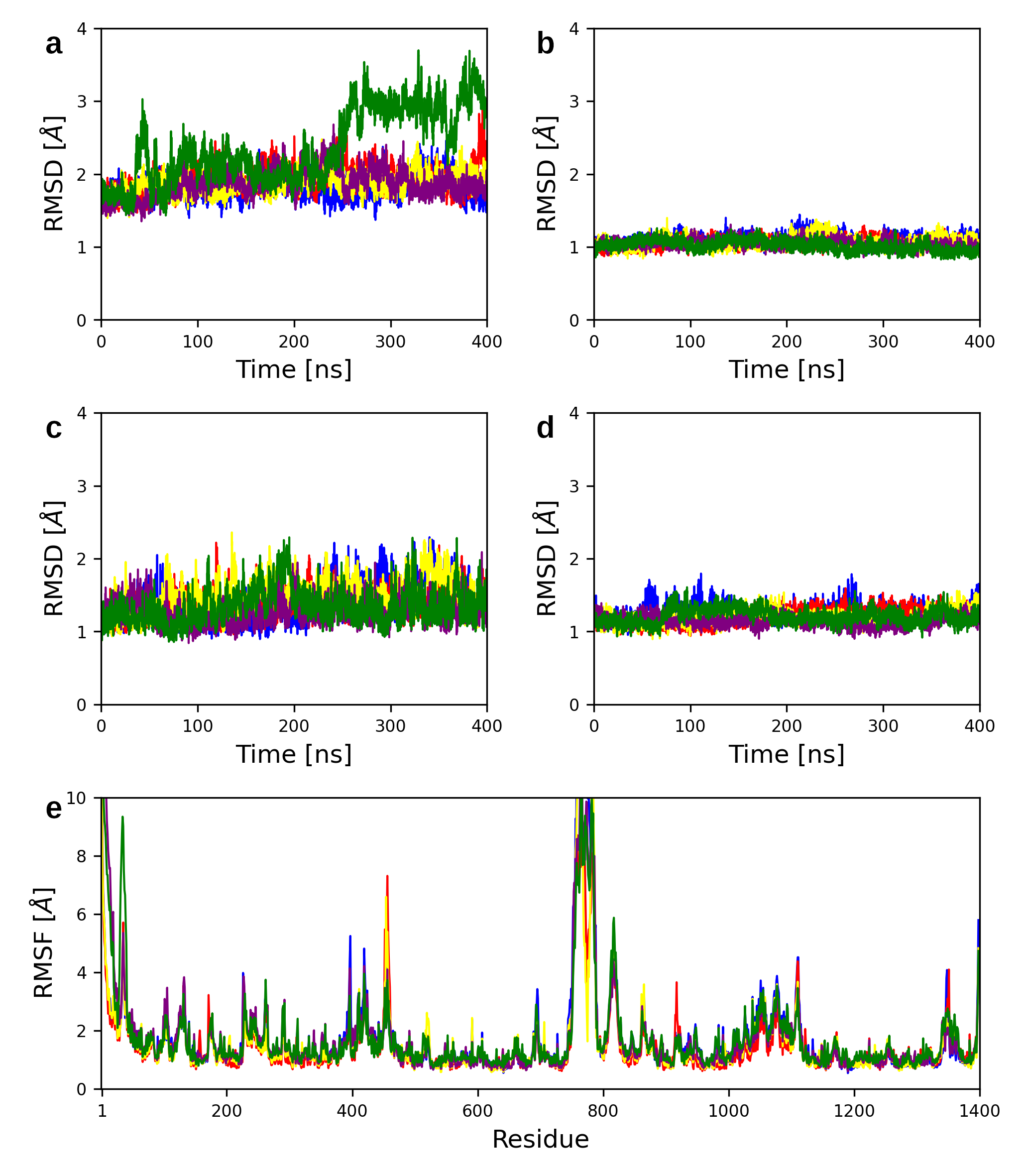


**Supplementary Figure 9:** **RMSD and RMSF of five independent replicas of MtABCG46.** **a-e** RMSD plots separated by regions: NBD1 (**a**), TMD1 (**b**), NBD2 (**c**) and TMD2 (**d**) and RMSF (**e**). Results show that TM regions remain more stable than NBD domains in all five repetitions.


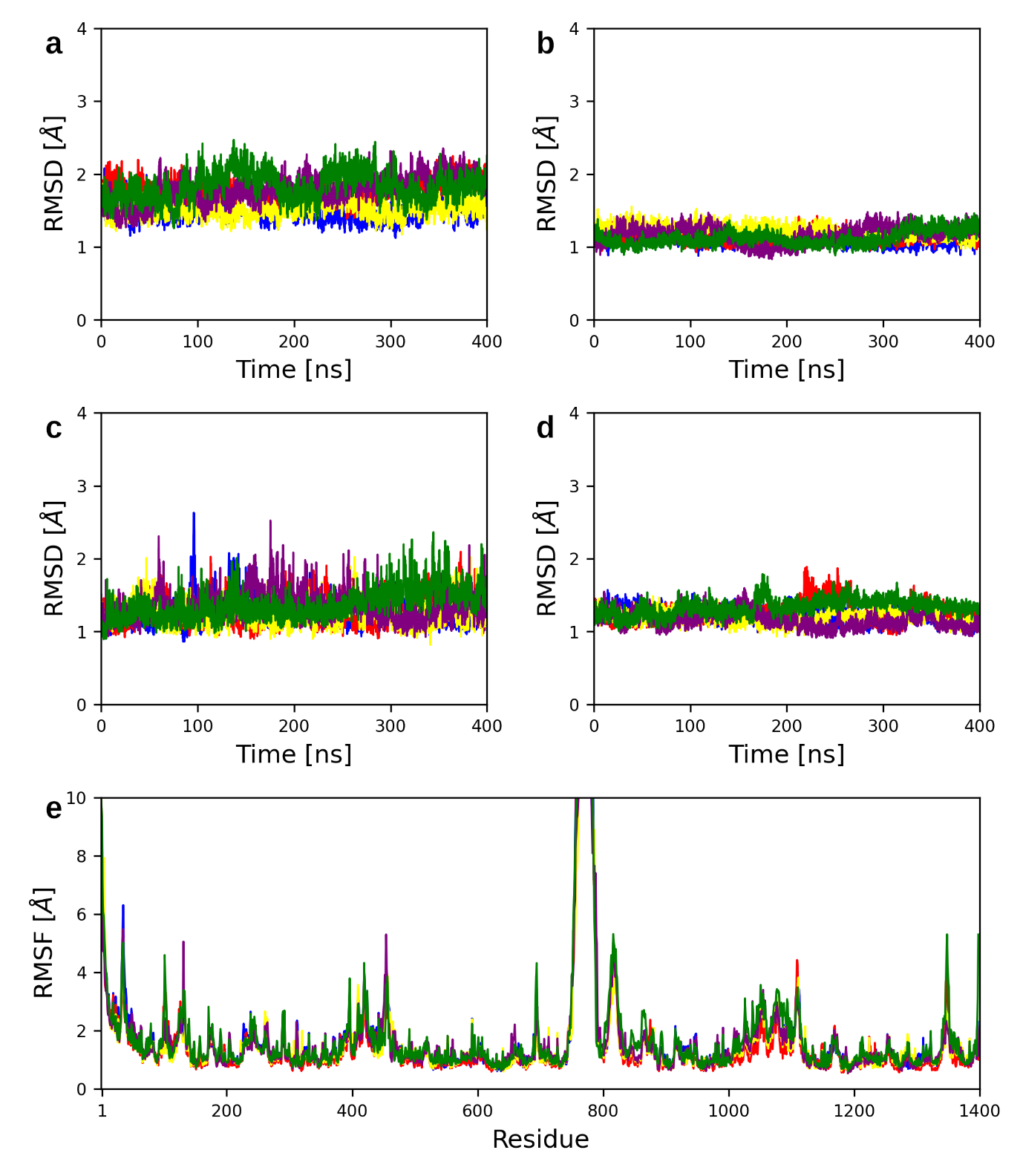


**Supplementary Figure 10: RMSD and RMSF of five independent replicas of MtABCG46 F562L mutant.** **a-e** RMSD plots separated by regions: NBD1 (**a**), TMD1 (**b**), NBD2 (**c**) and TMD2 (**d**) and RMSF (**e**). Results show that TM regions remain more stable than NBD domains in all five repetitions.


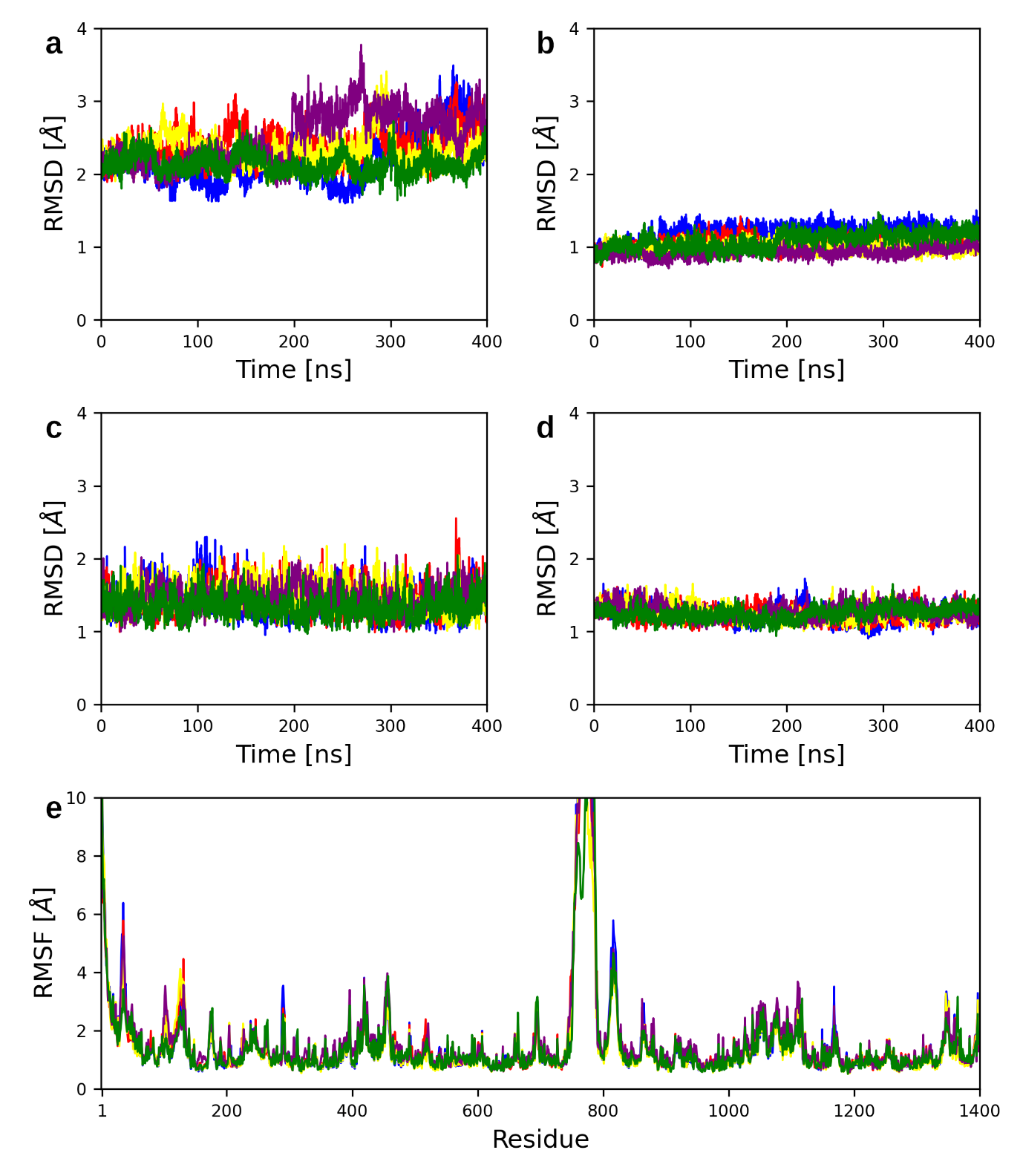


**Supplementary Figure 11: RMSD and RMSF of five independent replicas of MtABCG46 F562Y mutant.** **a-e** RMSD plots separated by regions: NBD1 (**a**), TMD1 (**b**), NBD2 (**c**) and TMD2 (**d**) and RMSF (**e**). Results show that TM regions remain more stable than NBD domains in all five repetitions.


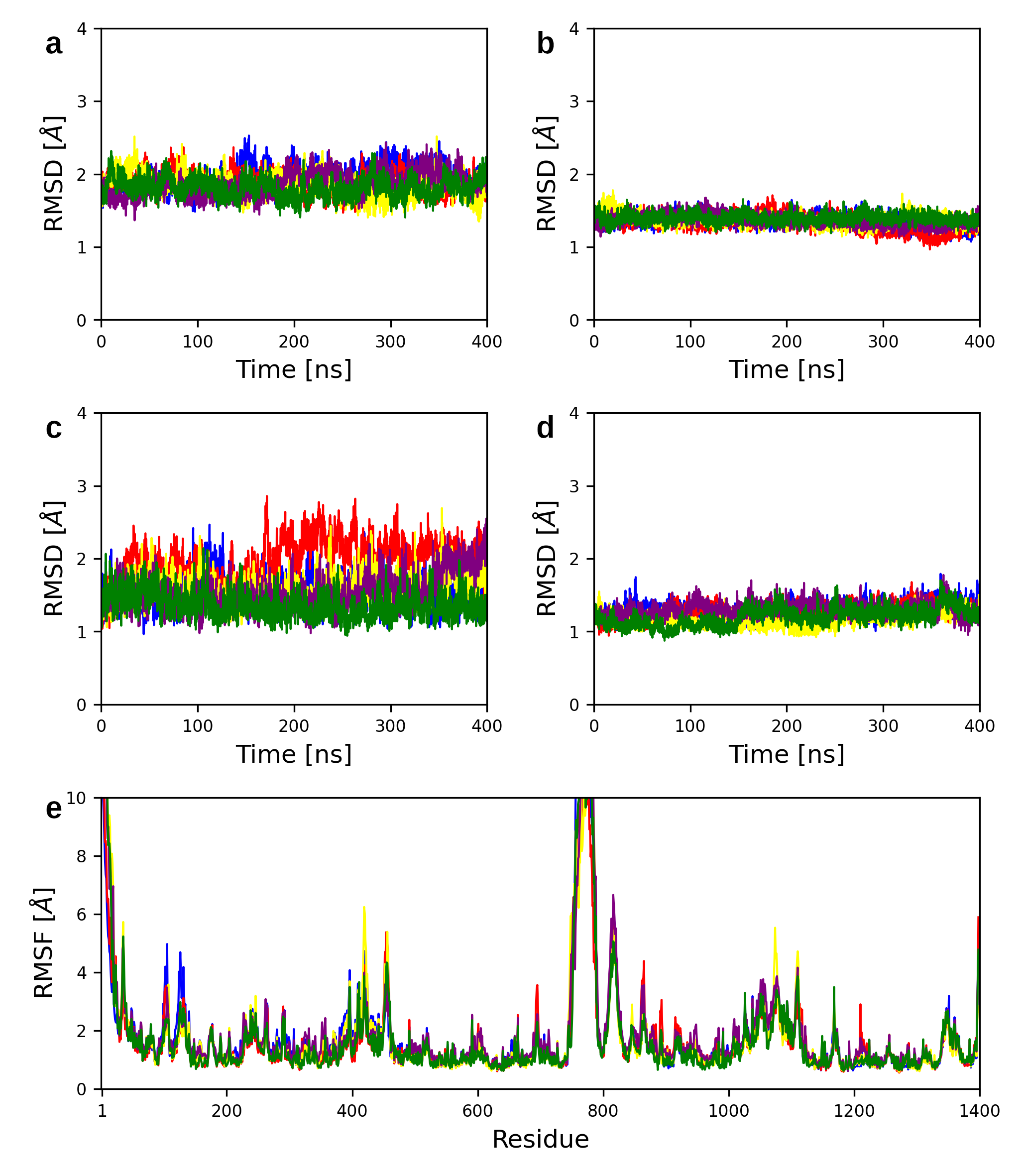


**Supplementary Figure 12: RMSD and RMSF of five independent replicas of MtABCG46 F562A mutant.** **a-e** RMSD plots separated by regions: NBD1 (**a**), TMD1 (**b**), NBD2 (**c**) and TMD2 (**d**) and RMSF (**e**). Results show that TM regions remain more stable than NBD domains in all five repetitions.


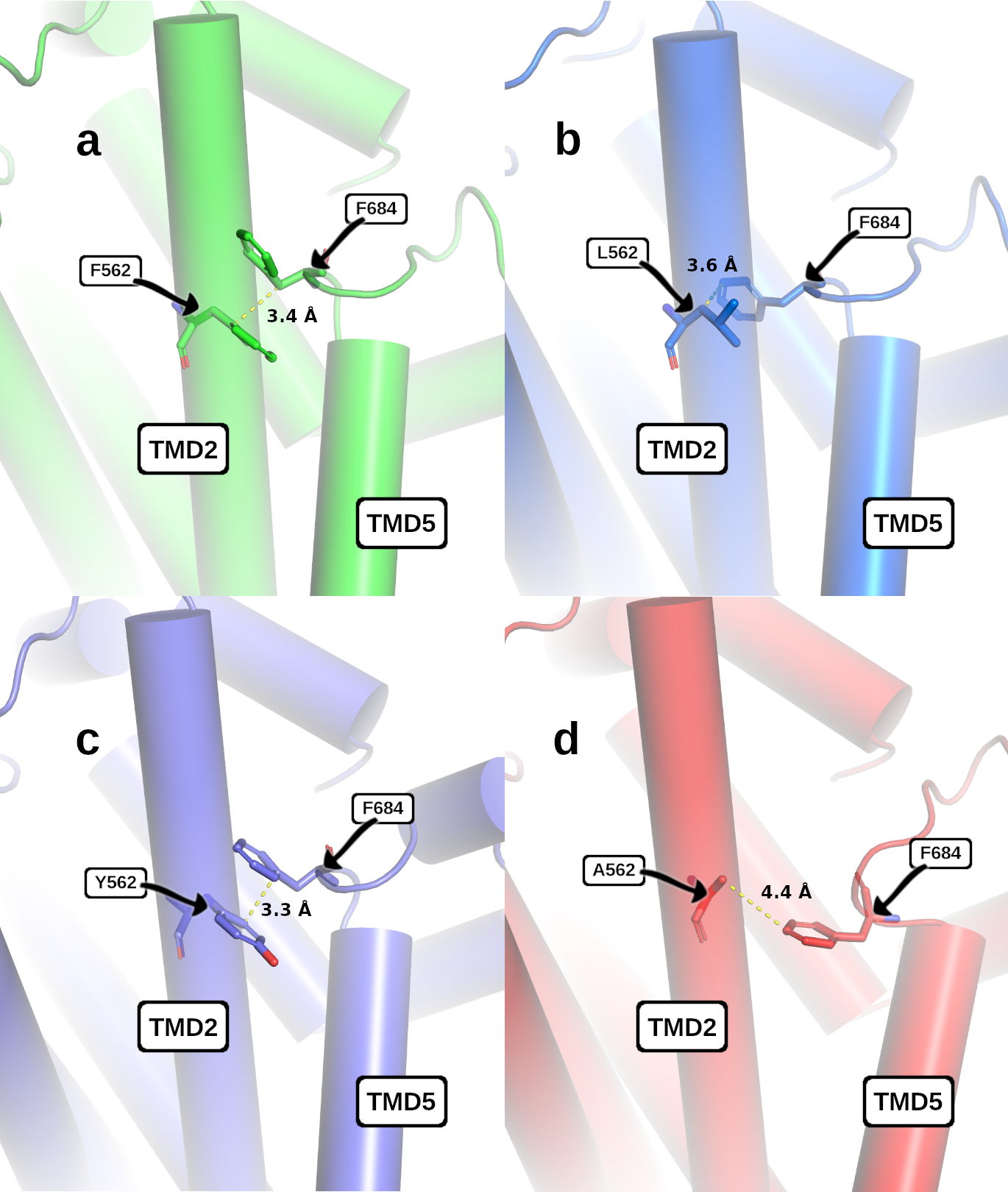


**Supplementary Figure 13: Parallel displaced π-stacking interaction between F684 and altered residues at position 562.** **a-d** Sliced trans-membrane lateral view of MtABCG46, showcasing the non-covalent contacts between F684 and altered residues (marked by arrows) as follows: F562 in WT (**a**), L562 in leucine mutant (**b**), Y562 in tyrosine mutant (**c**) and A562 in alanine mutant (**d**). The distances between the closest atoms of F684 and respective residues at position 562 are shown as dashed lines.


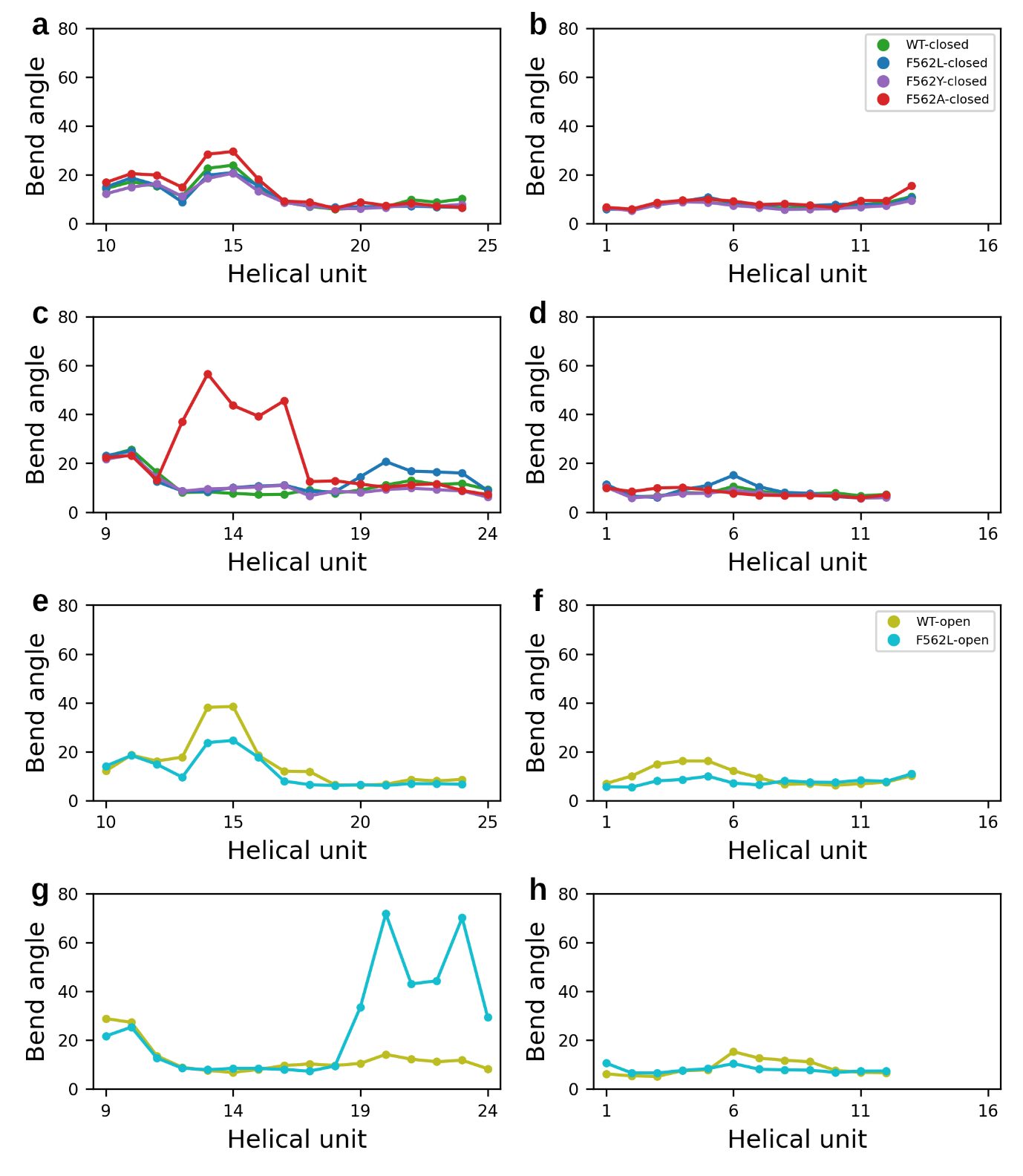


**Supplementary Figure 14: Bending angles of TMD helices forming the access path in MtABCG46 WT and mutants.** The angles (in degrees) for helical units defined by the HELANAL module of MDAnalysis are presented for: TMD helix 2 (**a**), TMD helix 5 (**b**), TMD helix 8 (**c**), and TMD helix 11 (**d**) in the closed states of MtABCG46 variants, and for TMD helix 2 (**e**), TMD helix 5 (**f**), TMD helix 8 (**g**), and TMD helix 11 (**h**) in the open states of MtABCG46 WT and F562L variants. Only the regions covering the entrance path are shown. The averages for five replications of each variant are shown as dots. The helical unit is defined as seven contiguous C-alpha atoms, for more details see: Sugeta H. & Miyazawa T. (1967). General method for calculating helical parameters of polymer chains from bond lengths, bond angles, and internal-rotation angles. *Biopolymers*. 5:673-679.


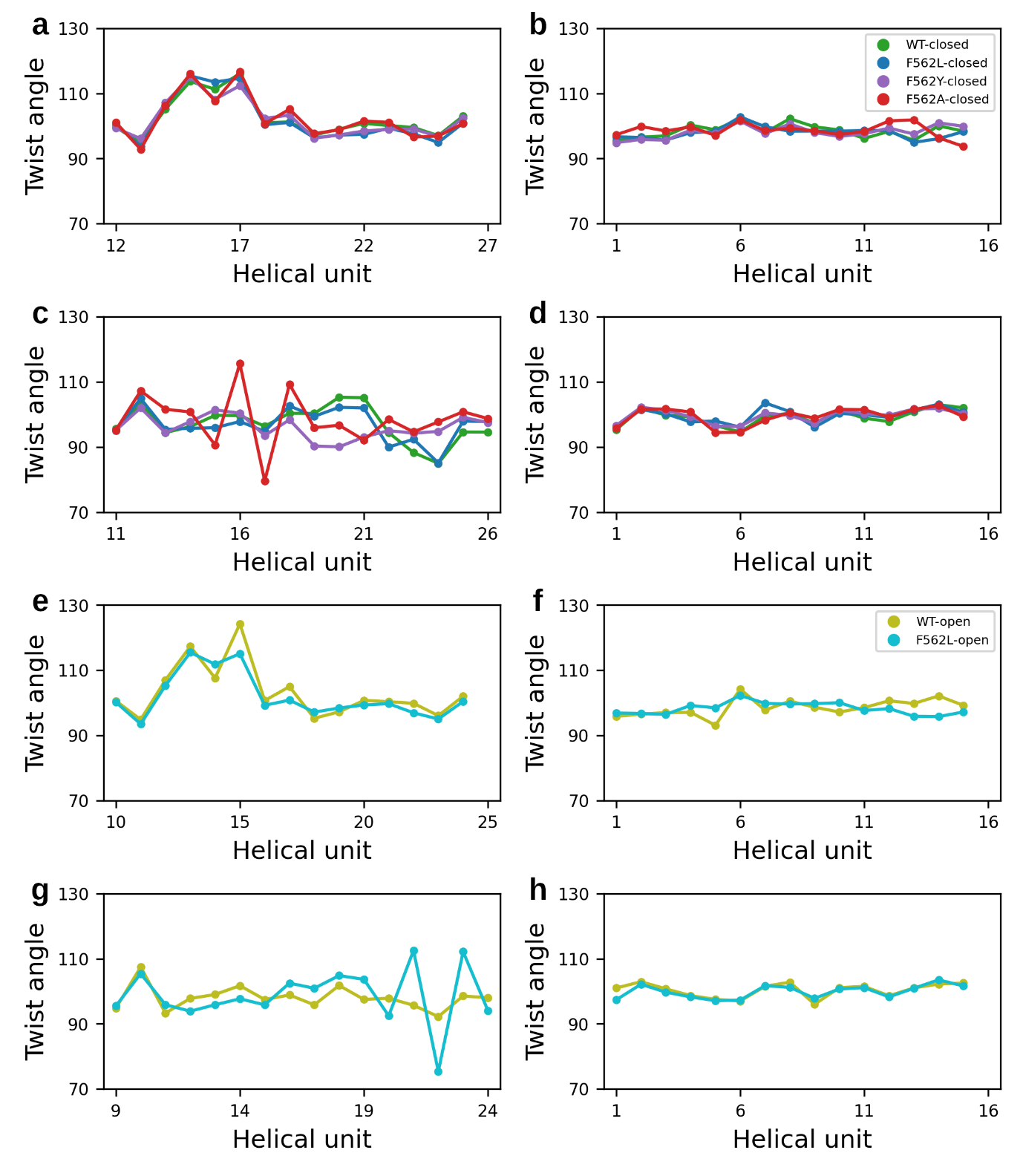


**Supplementary Figure 15: Twisting angles of TMD helices forming the access path in MtABCG46 WT and mutants.** The angles (in degrees) for helical units defined by the HELANAL module of MDAnalysis are presented for: TMD helix 2 (**a**), TMD helix 5 (**b**), TMD helix 8 (**c**), and TMD helix 11 (**d**) in the closed states of MtABCG46 variants, and for TMD helix 2 (**e**), TMD helix 5 (**f**), TMD helix 8 (**g**), and TMD helix 11 (**h**) in the open states of MtABCG46 WT and F562L variants. Only the regions covering the entrance path are shown. The averages for five replications of each variant are shown as dots. The helical unit is defined as four contiguous C-alpha atoms, for more details see: Sugeta H. & Miyazawa T. (1967). General method for calculating helical parameters of polymer chains from bond lengths, bond angles, and internal-rotation angles. *Biopolymers*. 5:673-679.


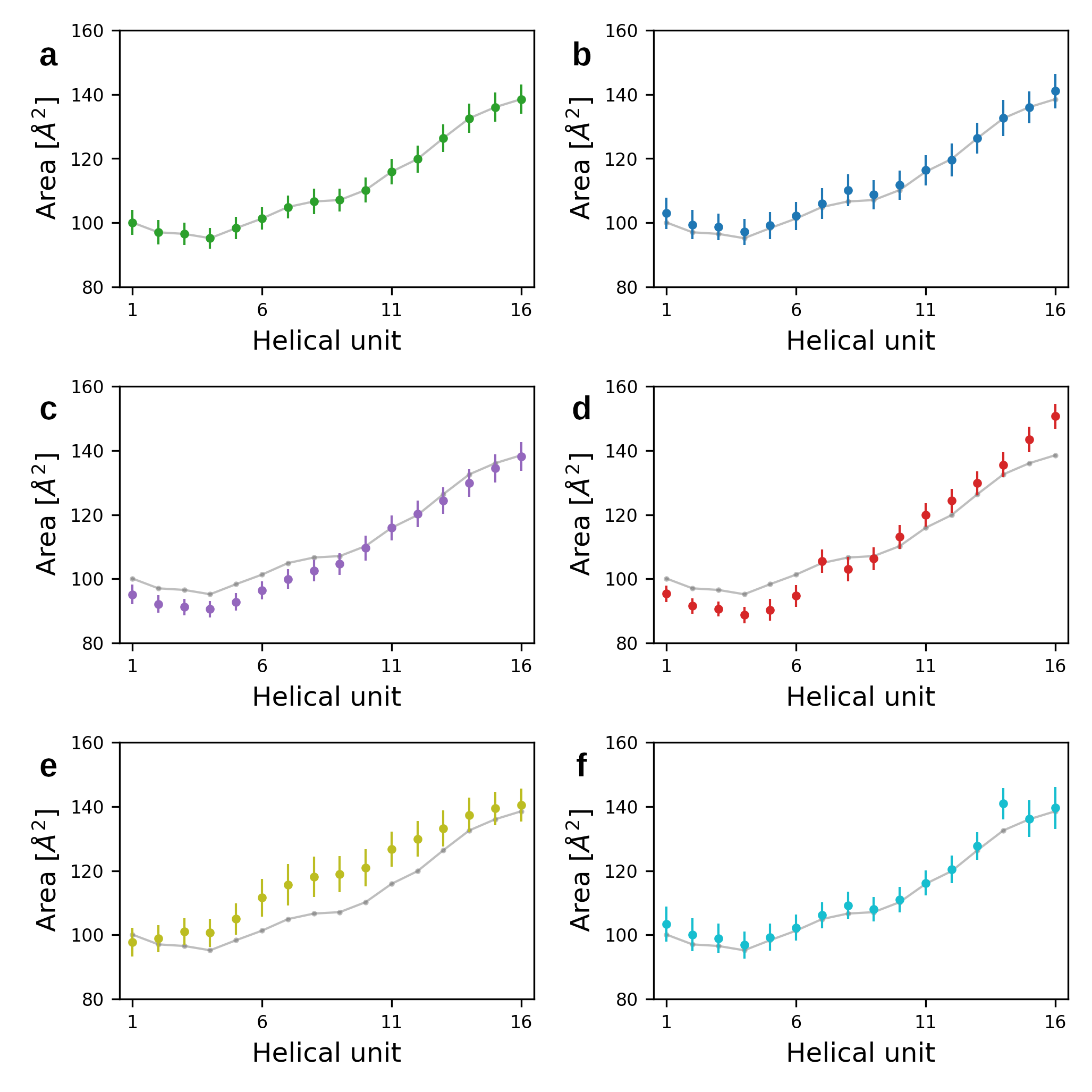


**Supplementary Figure 16: Area enclosed by TMD helices forming the access path in MtABCG46 IF closed and open states.** The areas calculated for each helical unit defined by the HELANAL module of MDAnalysis are presented. The entry to the access path (intra-cellular side) is at the left of the plot, with the deepest region at helical unit 16. Averages of five replicas for each variant are shown as dots with standard deviations for WT-closed (**a**), F562L-closed (**b**), F562Y-closed (**c**), F562A-closed (**d**), WT-open (**e**), and F562L-open (**f**). Shown in all plots as gray lines are the averages of the WT-closed state for comparison. The helical unit is defined as four contiguous C-alpha atoms, for more details see: Sugeta H. & Miyazawa T. (1967). General method for calculating helical parameters of polymer chains from bond lengths, bond angles, and internal-rotation angles. *Biopolymers*. 5:673-679.


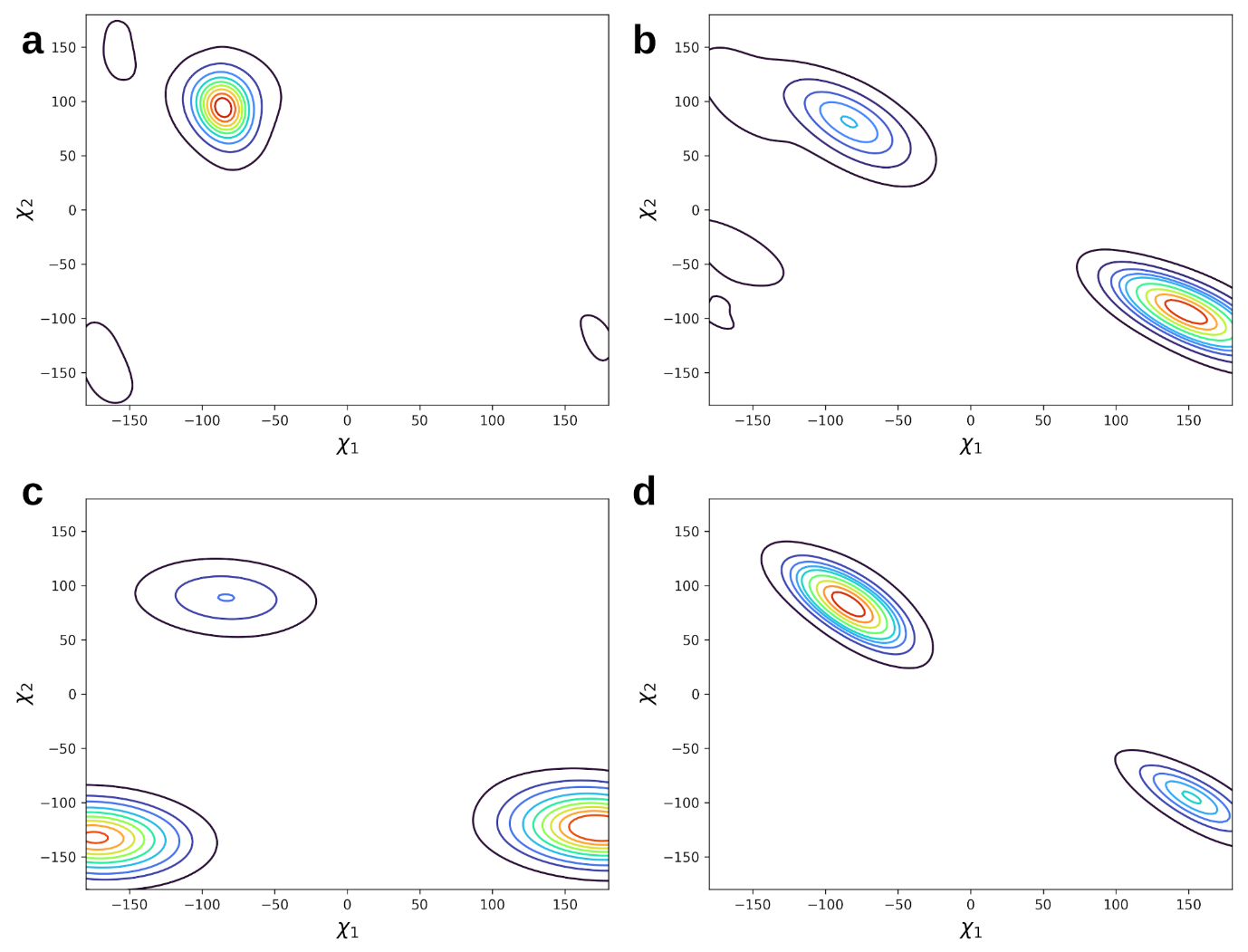


**Supplementary Figure 17:** **Conformational space adopted by N1331.** Kernel density estimate plots for the chi angles of N1331 in MtABCG46 variants WT (**a**), F562L (**b**), F562Y (**c**) and F562A (**d**) mutants.


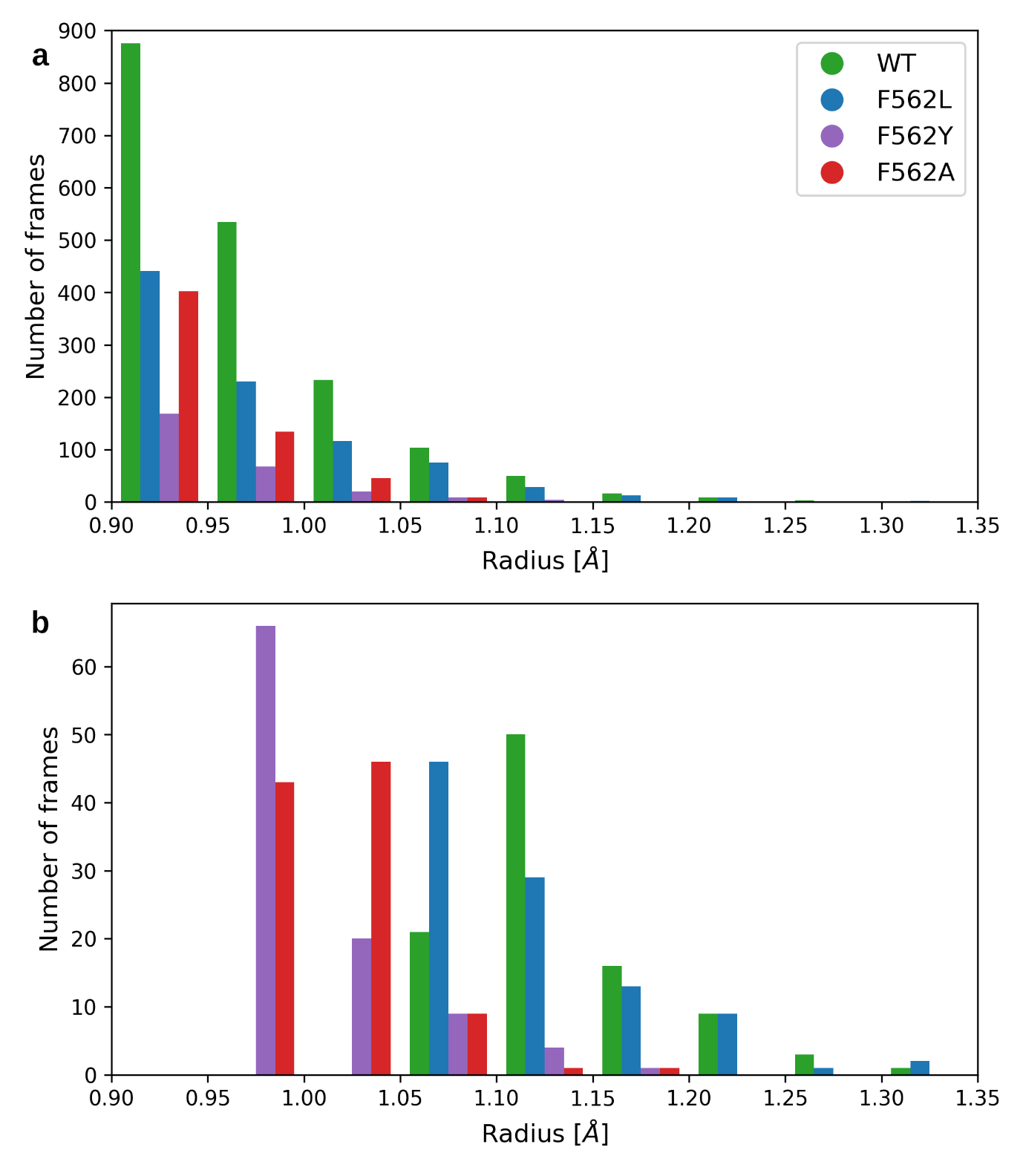


**Supplementary Figure 18: Presence of the access path in MtABCG46 WT and mutants.** The access path population obtained of 5 MD simulations of 400 ns per variant (same MDs presented in Supplementary Figures 9-12) is shown for all the paths present in each variant (**a**), and the 100 widest paths (**b**). WT results are the same as for Supplementary Figure 5.


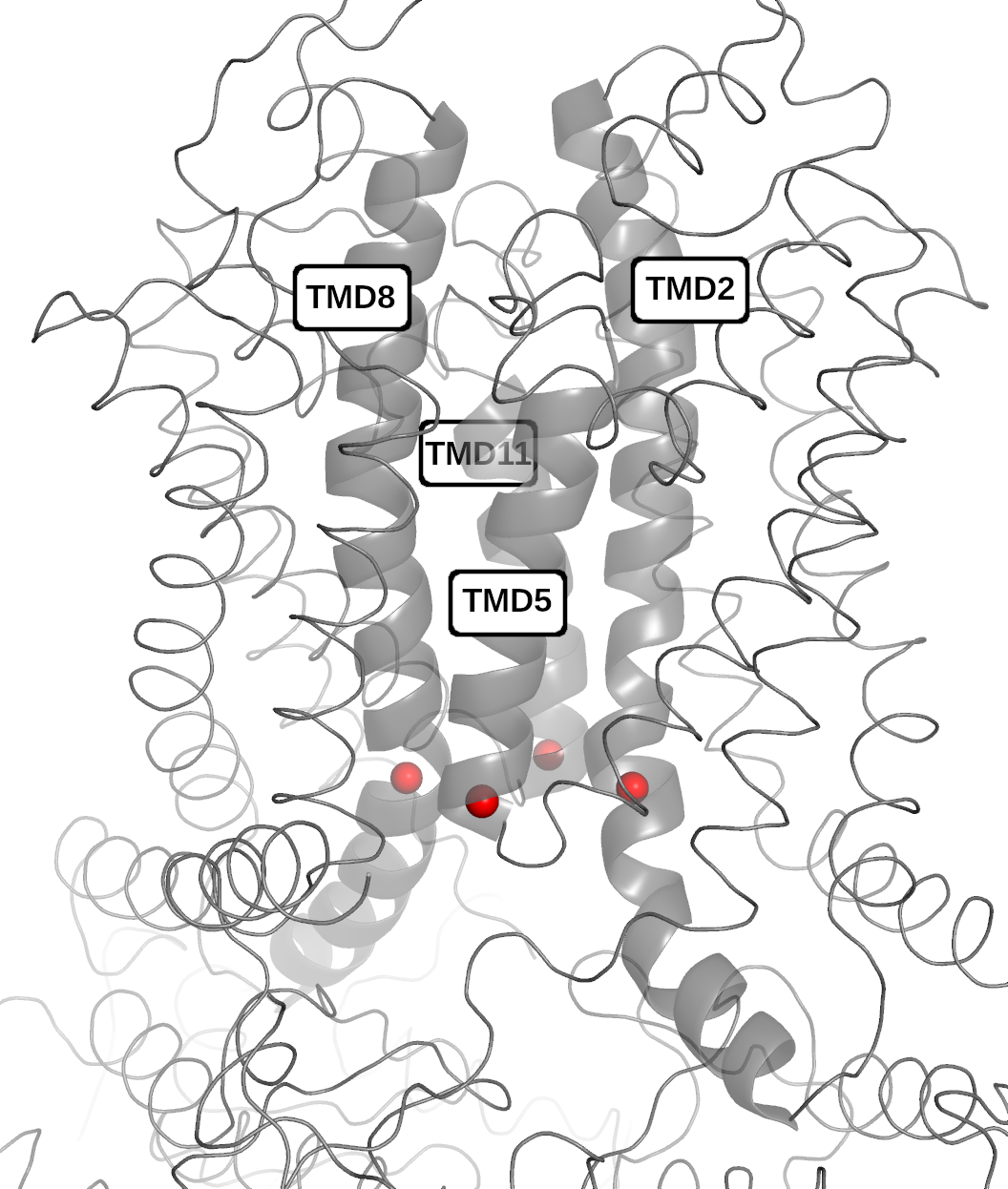


**Supplementary Figure 19:** **Area of the entrance to the access path in MtABCG46.** The four TMD helices 2, 5, 8, and 11 are represented as a cartoon, with the entry region delimited by the four red spheres corresponding to the helix centerlines defined by the HELANAL module of MDAnalysis.


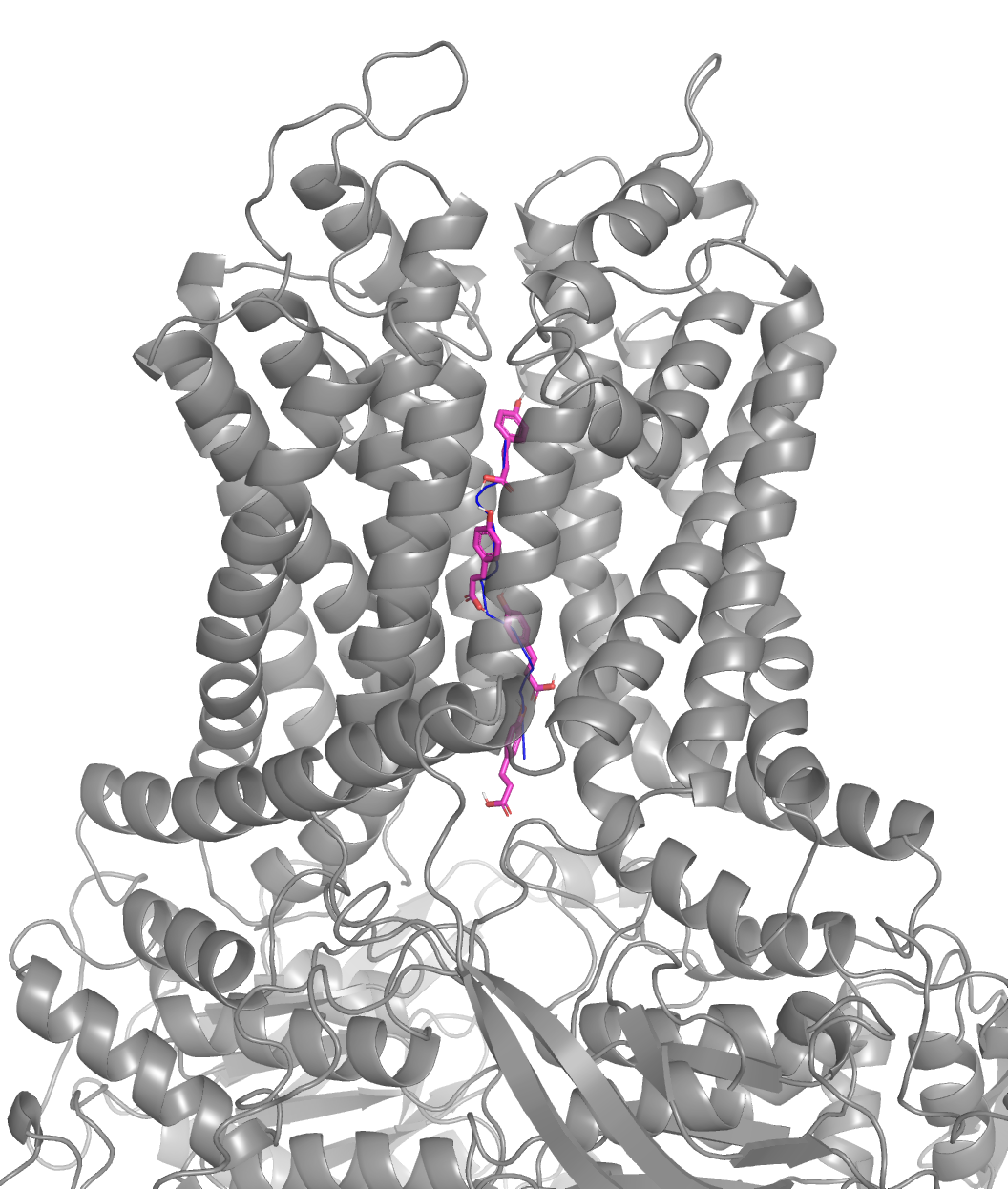


**Supplementary Figure 20: Migration from the intracellular region to the internal cavity in MtABCG46.** An example of the migration of 4-coumarate (magenta sticks) across the access path in MtABCG46, four regularly spaced snapshots of a CaverDock simulation are depicted along the path centerline (blue stick).


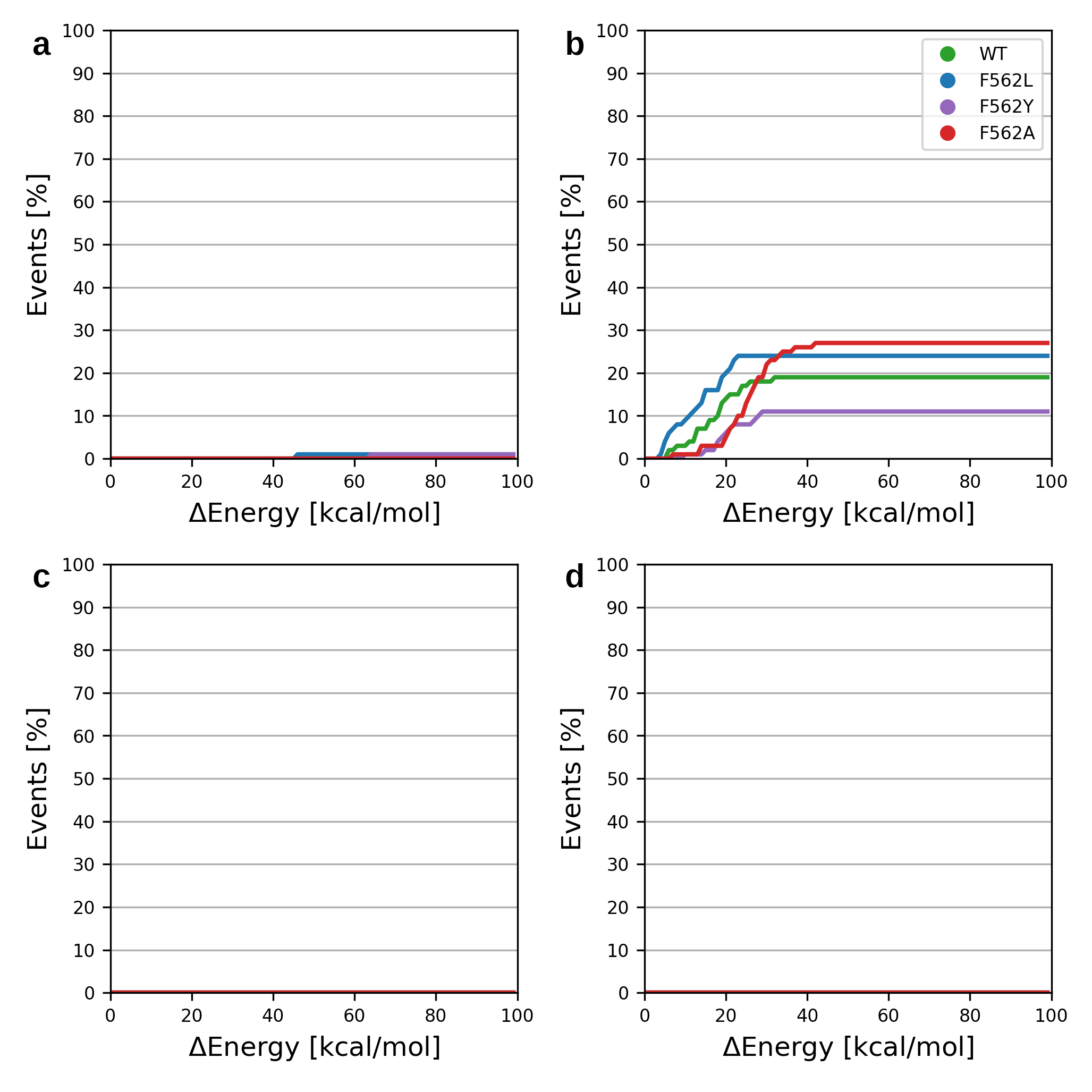


**Supplementary Figure 21: Successful migration events from the intracellular region to the internal cavity of MtABCG46 WT and mutants.** Cumulative distributions of energetic barriers for transport to the internal cavity with favorable binding for the widest 100 paths for liquiritigenin (**a**), charged form of 4-coumarate (**b**), 7,4’-dihydroxyflavone (**c**) and isoliquiritigenin (**d**). Curves obtained are colored for WT, F562L, F562Y and F562A in green, blue, purple and red respectively.


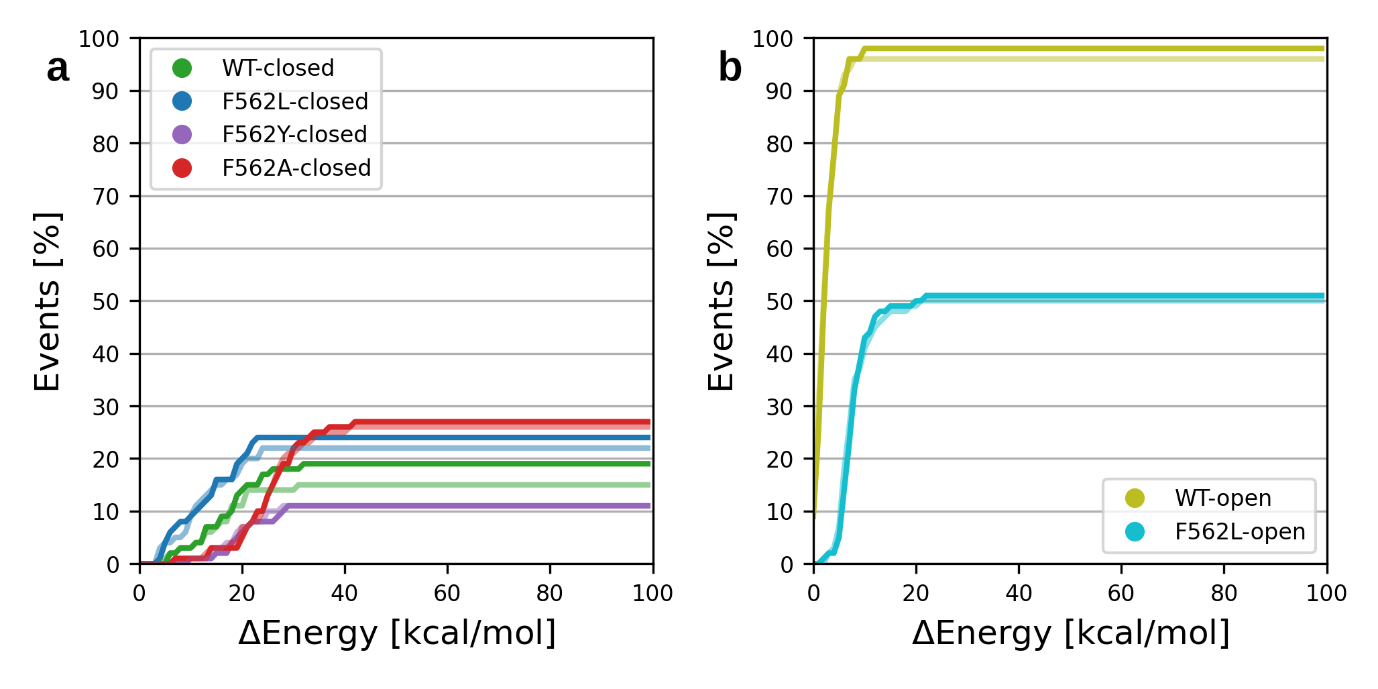


**Supplementary Figure 22: Successful migration events from the intracellular region to the internal cavity of MtABCG46 WT and mutants for 4-coumarate.** Cumulative distributions of energetic barriers for transport to the internal cavity with favorable binding for the widest 100 paths for WT, F562L, F562Y and F562A in IF-closed state (**a**), and only for WT and F562L in IF-open state (**b**). In **a** and **b**, the hard lines correspond to the charged form of 4-coumarate, and the faded lines to its neutral form.


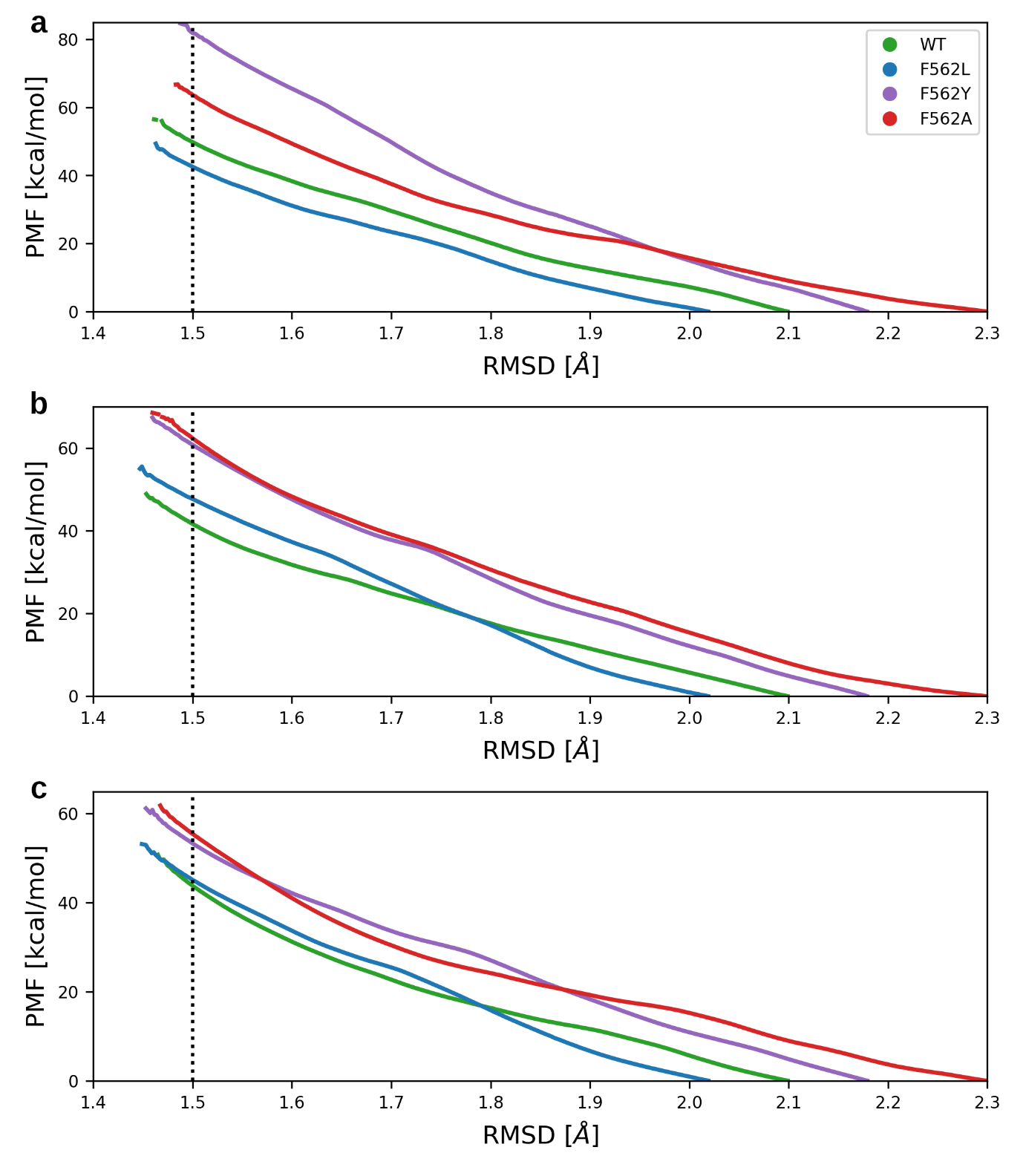


**Supplementary Figure 23: Potential of Mean Force (PMF) for US simulations of MtABCG46 WT and mutants.** PMF curves obtained with the WHAM method for 3 US simulations are presented for 1 ns (**a**), 2 ns (**b**) and 4 ns (**c**) of simulation time per umbrella for MtABCG46 WT and variants.


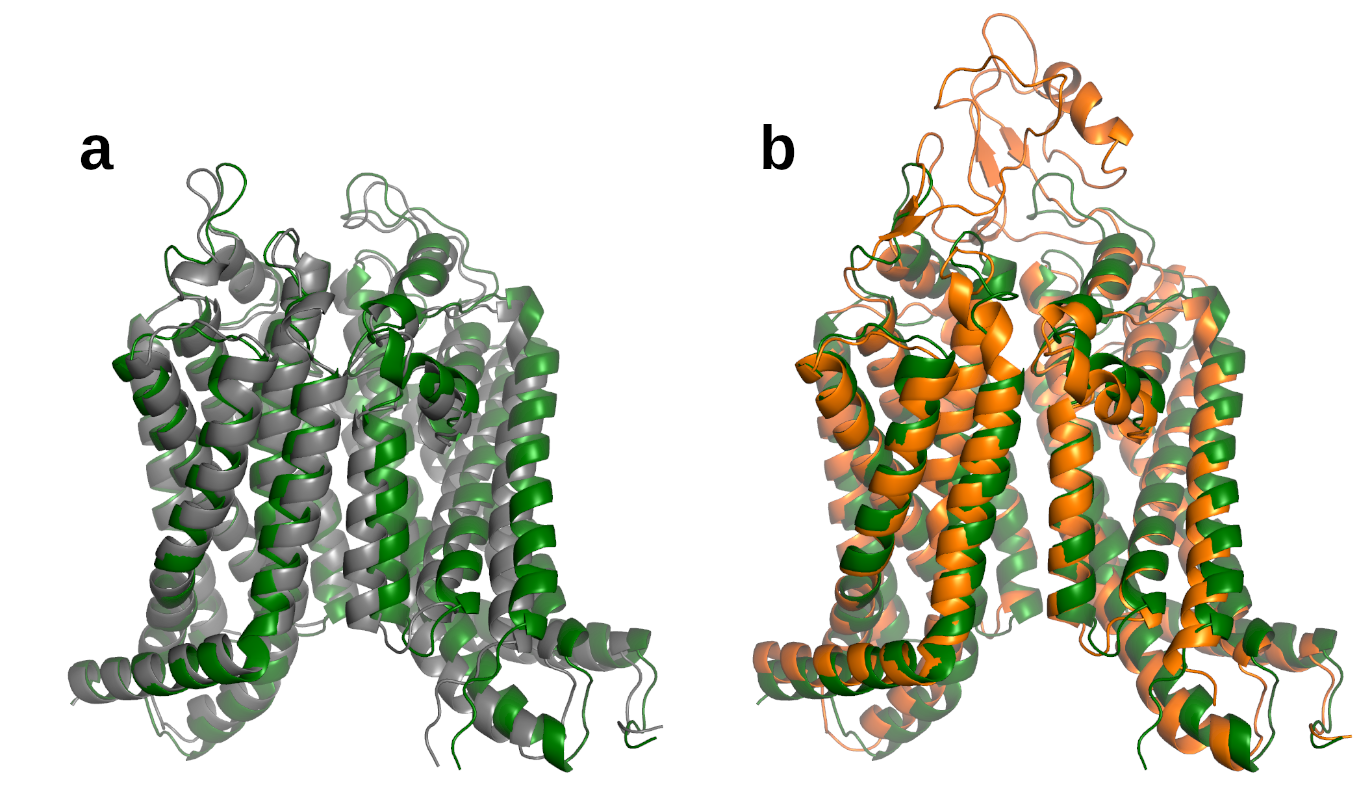


**Supplementary Figure 24: Structural comparison of MtABCG46 closed and open IF structures with ScPDR5.** TMD helices of MtABCG46 and ScPDR5 aligned: MtABCG46 in IF-closed state (gray, same structure as in Supplementary Figure 2) aligned to MtABCG46 IF-open state (dark green) (**a**), and MtABCG46 IF-open state aligned to ScPDR5 structure (orange) with Rhodamine 6G ligand bound (PDB ID: 7p05) (**b**).


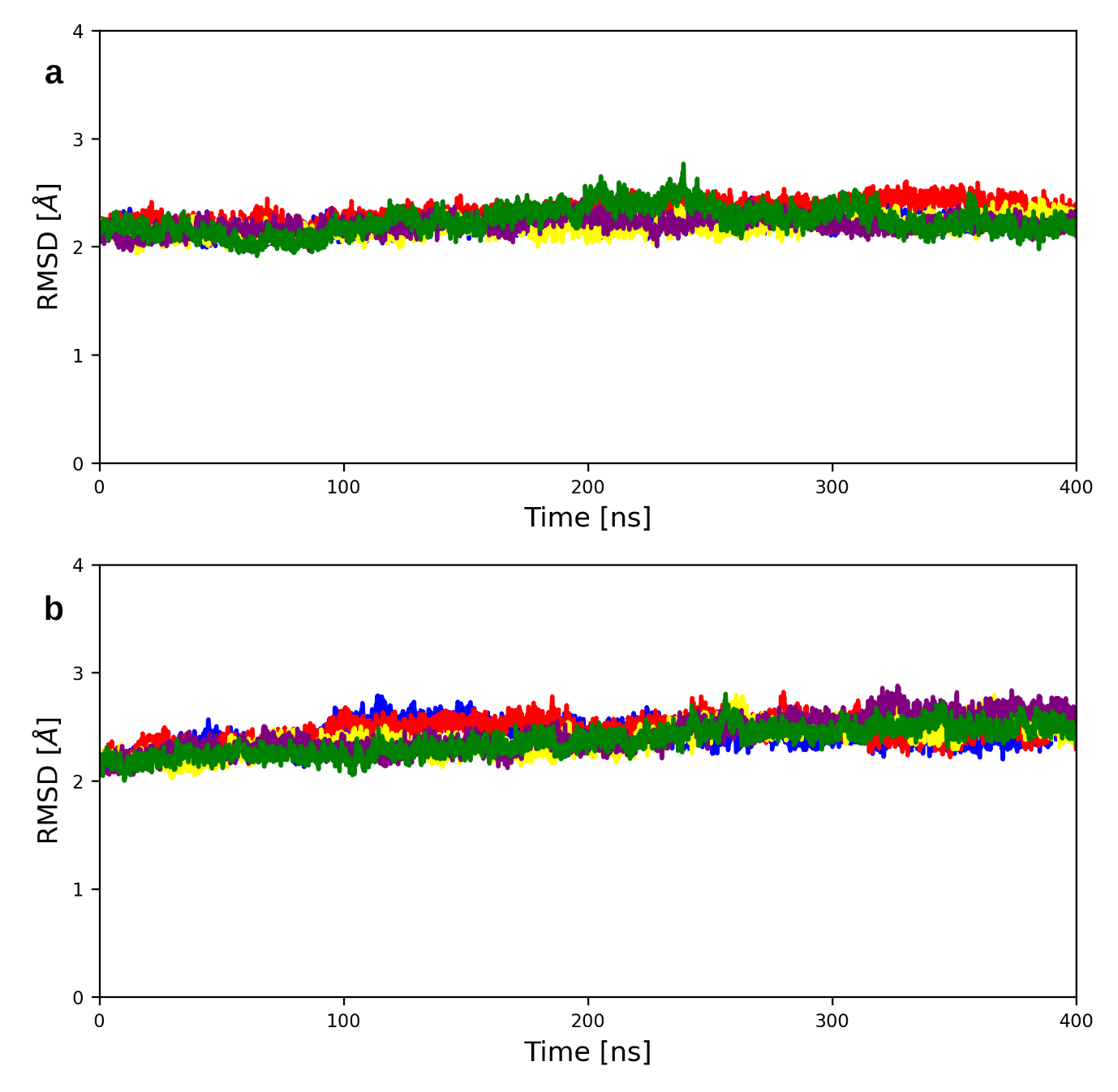


**Supplementary Figure 25: RMSD of five independent replicas of the TMD helices of MtABCG46 to ScPDR5 structure bound to R6 ligand.** RMSD of TMD helices of MtABCG46 WT in an IF-open conformation (**a**) and F562L mutant (**b**) to the TMD of ScPDR5 with Rhodamine 6G ligand bound (PDB ID: 7p05).


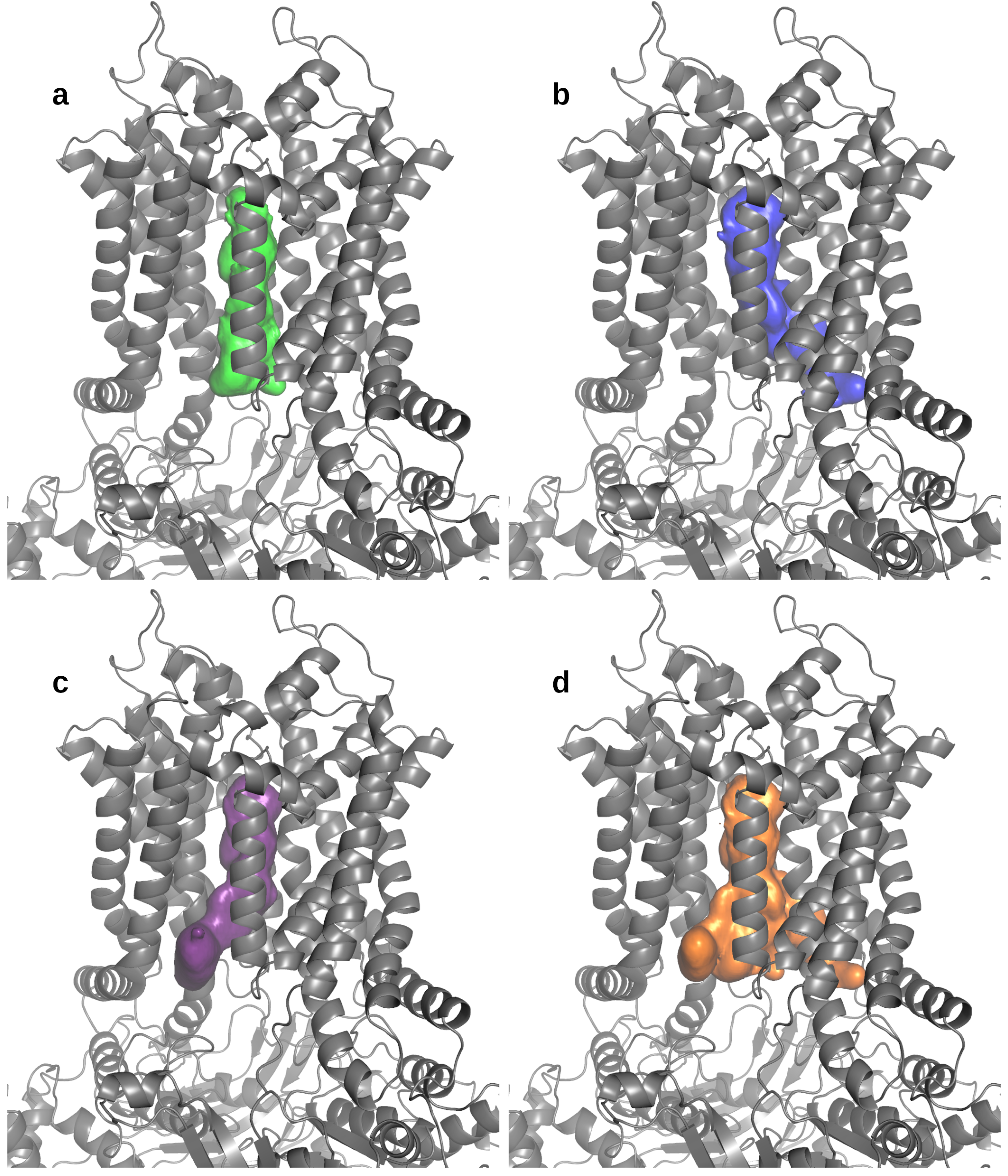


**Supplementary Figure 26: Access paths found in MtABCG46 IF-open state.** Three paths reaching the internal TMD cavity from the IC region are found in the IF-open state of MtABCG46 WT and F562L variant. Surface representation of tunnels 5 (**a**), 6 (**b**), 9 (**c**), and a merged view of all (**d**) are shown in green, blue, purple and orange respectively. The tunnels presented correspond to the data of Supplementary Table 5.


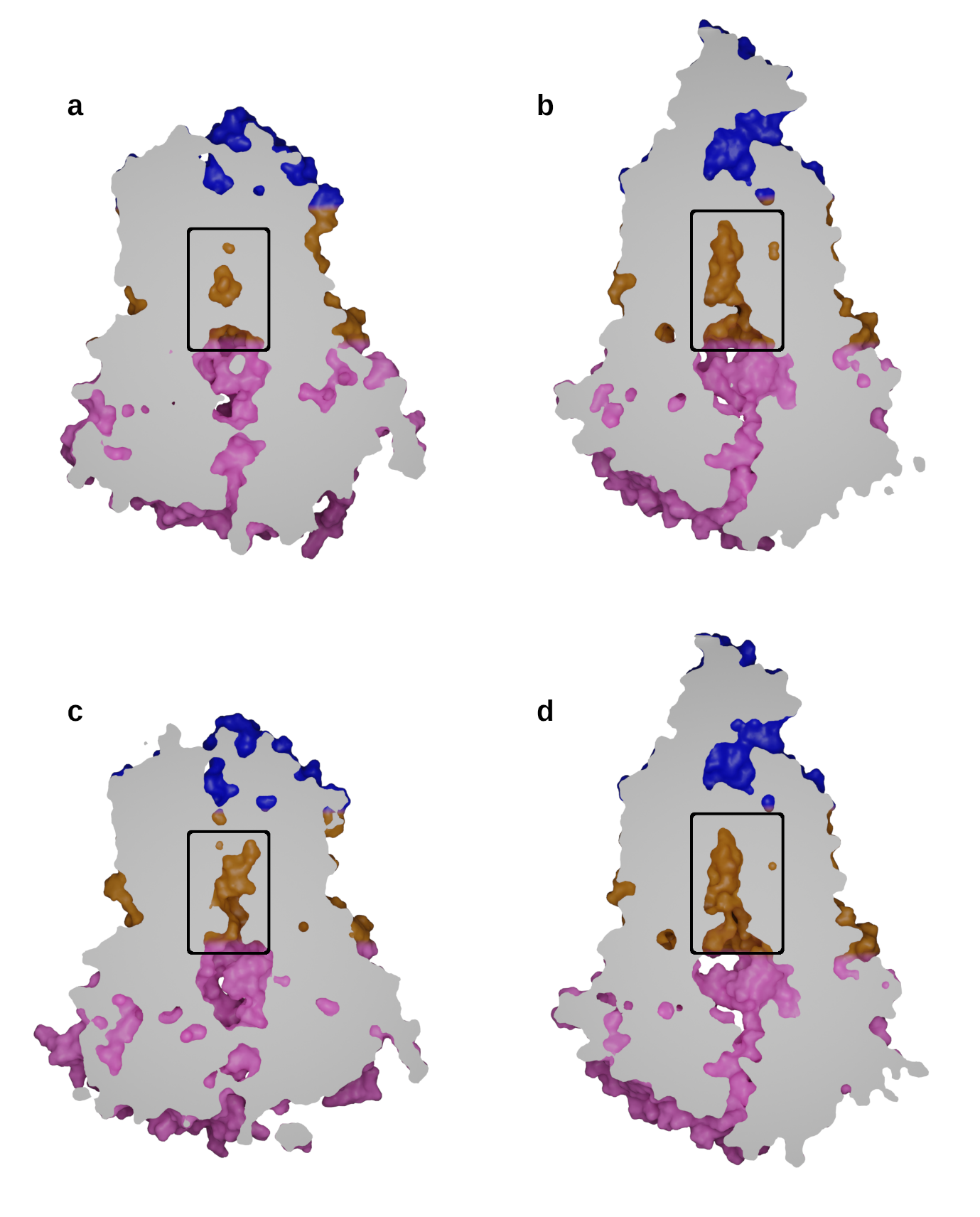


**Supplementary Figure 27: Central cavities of MtABCG46 and ScPDR5 in closed and open IF states.** Sliced surface side view of MtABCG46 in IF-closed (**a**) and IF-open (**c**) states, and ScPDR5 apo (**b**) and rhodamine-6G bound (**d**) states. Internal cavities and access paths are enclosed by black boxes.


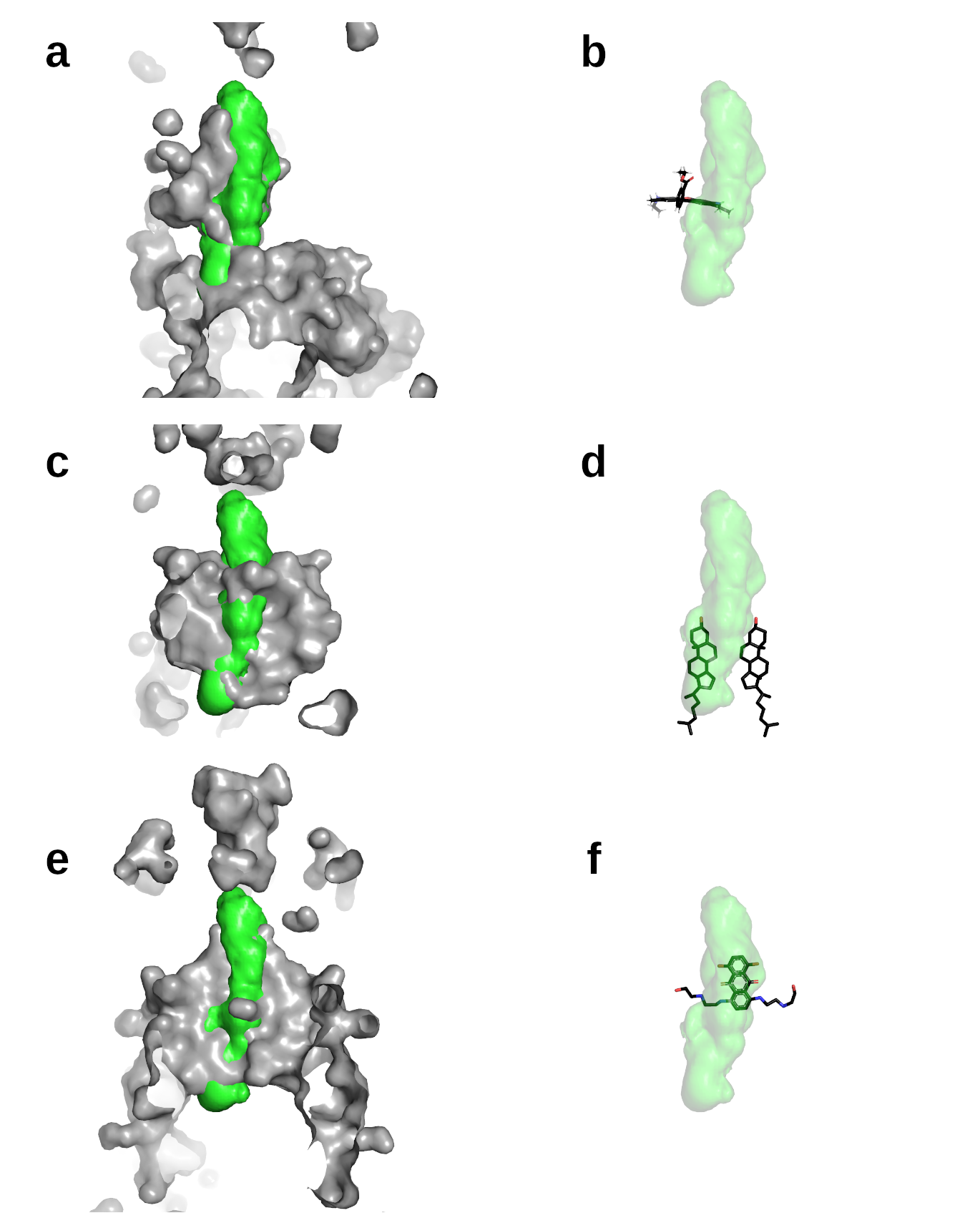


**Supplementary Figure 28: Cavities in related ABCG transporters and presence of ligands therein. a, c, e** Comparison between the access path ensemble in MtABCG46 shown as green surface and cavities found in experimental structures of related ABCG transporters shown as light gray surfaces: ScPDR5 (**a**), HsABCG1 (**c**), HsABCG2 (**e**). **b**, **d**, **f** Ligands present in related ABCG transporters superposed on the volume of the access path in MtABCG46 are shown as sticks: rhodamine 6G in ScPDR5 (**b**), cholesterol in HsABCG1 (**d**) mitoxantrone in HsABCG2 (**f**). All the structures were aligned with TM-align.

| **Supplementary Table 1. Details of computational protocols applied in individual stages of MD simulations.** | | | | | | | | |
| --- | --- | --- | --- | --- | --- | --- | --- | --- |
| MD stage | Simulation time [ns] | Temperature [K] | Pressure [bar] | Positional restraint energy [kcal/mol] | | | | Dihedral restraint energy [kcal/mol] |
|  |  |  |  | Protein | ATP | Mg^2+^ | Membrane | Membrane |
| Minimization | 5000 steps | - | - | 10.0 | 2.5 | 2.5 | 2.5 | 250.0 |
| NVT Equilibration 1 | 1 | 303.15 | - | 10.0 | 2.5 | 2.5 | 2.5 | 250.0 |
| NVT Equilibration 2 | 1 | 303.15 | - | 5.0 | 2.5 | 2.5 | 2.5 | 100.0 |
| NPT Equilibration 3 | 1 | 303.15 | 1 | 2.5 | 2.5 | 2.5 | 1.0 | 50.0 |
| NPT Equilibration 4 | 1 | 303.15 | 1 | 1.0 | 1.0 | 1.0 | 0.5 | 50.0 |
| NPT Equilibration 5 | 1 | 303.15 | 1 | 0.5 | 0.5 | 0.5 | 0.1 | 25.0 |
| NPT Equilibration 6 | 1 | 303.15 | 1 | 0.1 | 0.5 | 0.5 | - | - |
| NPT Long equilibration | 100 | 303.15 | 1 | - | 0.05 | 0.05 | - | - |
| NPT Unrestrained equilibration | 100 | 303.15 | 1 | - | - | - | - | - |
| NPT Production | 400 | 303.15 | 1 | - | - | - | - | - |

| **Supplementary Table 2. MtABCG46 domains defined and residues used to calculate RMSD** | | |
| --- | --- | --- |
| Region | Residue range | RMSD range |
| NBD1 | M1 – L499 | T52 – L478 |
| TMD1 | N500 – L770 | K501 – V769 |
| Linker | G771 - L822 | n/a |
| NBD2 | P823 – P1145 | E825 – V1134 |
| TMD2 | T1146 – R1428 | S1151 – N1424 |

| **Supplementary Table 3. Presence of access path among MtABCG46 WT and mutants** | | | |
| --- | --- | --- | --- |
| **Variant** | **% of presence** | **Bottleneck radius** | |
|  |  | **Average** | **StDev** |
| WT | 9.08% | 0.97 | 0.06 |
| F562L | 4.49% | 0.98 | 0.07 |
| F562Y | 1.25% | 0.95 | 0.05 |
| F562A | 2.97% | 0.94 | 0.04 |

| **Supplementary Table 4. Top ten access path ensembles identified with TransportTools from five simulations of MtABCG46** | | | | | | | | | | |
| --- | --- | --- | --- | --- | --- | --- | --- | --- | --- | --- |
| **ID** | **MD runs** | **Total Frames** | **Avg BR ±StDev** | **Max BR** | **Avg Length ±StDev** | **Avg Curv ±StDev** | **Avg Throughput ±StDev** | **Priority** | **Exit region** | **Frequency** |
| 1 | 4 | 2290 | 1.10 ±0.19 | 2.06 | 24.76 ±2.91 | 1.27 ±0.14 | 0.49 ±0.09 | 0.223 | EC | 11.45% |
| 2 | 5 | 1402 | 1.03 ±0.13 | 1.72 | 28.90 ±4.75 | 1.39 ±0.13 | 0.39 ±0.08 | 0.135 | EC | 7.01% |
| **3** | **5** | **1816** | **0.97 ±0.06** | **1.32** | **34.58 ±2.94** | **1.33 ±0.09** | **0.19 ±0.04** | **0.087** | **IC** | **9.08%** |
| 4 | 3 | 1540 | 1.09 ±0.17 | 1.9 | 32.27 ±4.88 | 1.63 ±0.19 | 0.38 ±0.08 | 0.087 | EC | 7.70% |
| 5 | 4 | 938 | 1.07 ±0.15 | 1.7 | 26.82 ±5.32 | 1.36 ±0.22 | 0.39 ±0.10 | 0.073 | EC | 4.69% |
| 6 | 4 | 754 | 1.03 ±0.13 | 1.65 | 27.41 ±4.28 | 1.28 ±0.12 | 0.40 ±0.09 | 0.061 | EC | 3.77% |
| 7 | 3 | 995 | 1.07 ±0.16 | 1.71 | 35.45 ±4.55 | 1.71 ±0.17 | 0.30 ±0.07 | 0.045 | EC | 4.98% |
| 8 | 5 | 637 | 0.97 ±0.06 | 1.26 | 38.26 ±4.11 | 1.44 ±0.17 | 0.17 ±0.05 | 0.026 | IC | 3.19% |
| 9 | 2 | 734 | 1.04 ±0.14 | 1.55 | 30.46 ±4.43 | 1.57 ±0.18 | 0.35 ±0.08 | 0.025 | EC | 3.67% |
| 10 | 1 | 851 | 1.11 ±0.18 | 2.12 | 25.92 ±3.26 | 1.24 ±0.10 | 0.48 ±0.09 | 0.02 | EC | 4.26% |

MD runs – number of simulations in which the path ensemble was observed; Total Frames – number of frames in which the path ensemble was present; Avg BR ±StDev – average bottleneck radius of paths in the path ensemble with respective standard deviations; Max BR – maximal bottleneck radius of the widest path in the path ensemble, Avg Length ±StDev – average length of paths in the path ensemble with respective standard deviations; Avg Curv ±StDev – average curvature of paths in the path ensemble with respective standard deviations; Avg Throughput ±StDev – average throughput of paths in the path ensemble with respective standard deviations; Priority – priority of path ensemble; Exit region – location of the exit/entry of the path ensemble – IC,EC, and TM stand for intracellular, extracellular and transmembrane, respectively; Frequency – in how many frames across all analyzed input frames is the path ensemble present.

| **Supplementary Table 5. Top ten access path ensembles identified with TransportTools from simulations of MtABCG46 WT and F562L in IF-open states.** | | | | | | | | | | |
| --- | --- | --- | --- | --- | --- | --- | --- | --- | --- | --- |
| **WT-open** | | | | | | | | | | |
| **ID** | **MD runs** | **Total Frames** | **Avg BR ±StDev** | **Max BR** | **Avg Length ±StDev** | **Avg Curv ±StDev** | **Avg Throughput ±StDev** | **Priority** | **Exit region** | **Frequency** |
| 1 | 4 | 972 | 1.00 ±0.10 | 1.48 | 27.16 ±3.79 | 1.39 ±0.17 | 0.37 ±0.08 | 0.09 | EC | 6.08% |
| 2 | 4 | 853 | 1.01 ±0.11 | 1.51 | 27.12 ±3.27 | 1.23 ±0.11 | 0.41 ±0.08 | 0.088 | EC | 5.33% |
| 3 | 4 | 5831 | 1.04 ±0.12 | 1.71 | 23.94 ±4.03 | 1.40 ±0.20 | 0.36 ±0.08 | 0.522 | TM | 36.44% |
| 4 | 4 | 3873 | 0.99 ±0.08 | 1.37 | 26.62 ±4.32 | 1.47 ±0.20 | 0.32 ±0.07 | 0.309 | TM | 24.21% |
| **5** | **3** | **659** | **1.07 ±0.17** | **1.71** | **32.15 ±3.64** | **1.31 ±0.22** | **0.30 ±0.09** | **0.037** | **IC** | **4.12%** |
| **6** | **4** | **5071** | **1.02 ±0.11** | **1.69** | **36.09 ±4.00** | **1.41 ±0.11** | **0.26 ±0.06** | **0.325** | **IC** | **31.69%** |
| 7 | 3 | 2370 | 1.06 ±0.12 | 1.53 | 32.30 ±3.46 | 1.39 ±0.12 | 0.29 ±0.06 | 0.131 | TM/IC | 14.81% |
| 8 | 1 | 36 | 0.99 ±0.07 | 1.2 | 27.41 ±2.95 | 1.37 ±0.13 | 0.35 ±0.06 | 0.001 | EC | 0.23% |
| **9** | **3** | **1319** | **1.03 ±0.10** | **1.54** | **35.22 ±2.99** | **1.39 ±0.12** | **0.25 ±0.05** | **0.063** | **IC** | **8.24%** |
| 10 | 4 | 2221 | 1.04 ±0.12 | 1.59 | 34.54 ±3.45 | 1.39 ±0.17 | 0.30 ±0.07 | 0.166 | TM/IC | 13.88% |
| **F562L-open** | | | | | | | | | | |
| **ID** | **MD runs** | **Total Frames** | **Avg BR ±StDev** | **Max BR** | **Avg Length ±StDev** | **Avg Curv ±StDev** | **Avg Throughput ±StDev** | **Priority** | **Exit region** | **Frequency** |
| 1 | 5 | 3912 | 1.10 ±0.20 | 1.9 | 25.11 ±3.28 | 1.31 ±0.08 | 0.44 ±0.11 | 0.432 | EC | 19.56% |
| 2 | 4 | 2170 | 1.06 ±0.20 | 2.04 | 25.77 ±3.62 | 1.21 ±0.08 | 0.43 ±0.10 | 0.188 | EC | 10.85% |
| 3 | n/a | n/a | n/a | n/a | n/a | n/a | n/a | n/a | n/a | n/a |
| 4 | 2 | 210 | 0.99 ±0.09 | 1.34 | 34.96 ±3.77 | 2.04 ±0.21 | 0.21 ±0.06 | 0.004 | TM | 1.05% |
| **5** | **5** | **2555** | **0.99 ±0.09** | **1.46** | **31.69 ±2.95** | **1.30 ±0.08** | **0.24 ±0.06** | **0.154** | **IC** | **12.78%** |
| **6** | **n/a** | **n/a** | **n/a** | **n/a** | **n/a** | **n/a** | **n/a** | **n/a** | **n/a** | **n/a** |
| 7 | 5 | 516 | 0.99 ±0.09 | 1.31 | 37.43 ±3.62 | 1.58 ±0.15 | 0.19 ±0.06 | 0.024 | TM/IC | 2.58% |
| 8 | 5 | 2299 | 1.13 ±0.20 | 1.98 | 27.26 ±6.34 | 1.40 ±0.27 | 0.41 ±0.12 | 0.238 | EC | 11.50% |
| **9** | **5** | **1399** | **0.99 ±0.09** | **1.39** | **34.48 ±4.06** | **1.38 ±0.14** | **0.23 ±0.07** | **0.079** | **IC** | **7.00%** |
| 10 | n/a | n/a | n/a | n/a | n/a | n/a | n/a | n/a | n/a | n/a |

MD runs – number of simulations in which the path ensemble was observed; Total Frames – number of frames in which the path ensemble was present; Avg BR ±StDev – average bottleneck radius of paths in the path ensemble with respective standard deviations; Max BR – maximal bottleneck radius of the widest path in the path ensemble, Avg Length ±StDev – average length of paths in the path ensemble with respective standard deviations; Avg Curv ±StDev – average curvature of paths in the path ensemble with respective standard deviations; Avg Throughput ±StDev – average throughput of paths in the path ensemble with respective standard deviations; Priority – priority of path ensemble; Exit region – location of the exit/entry of the path ensemble – IC,EC, and TM stand for intracellular, extracellular and transmembrane, respectively; Frequency – in how many frames across all analyzed input frames is the path ensemble present. n/a – corresponding path ensemble was absent in F562L simulations.
